# Associations Between Genetic Risk for Adult Suicide Attempt and Suicidal Behaviors in Young Children in the US

**DOI:** 10.1001/jamapsychiatry.2022.2379

**Published:** 2022-08-31

**Authors:** Phil H. Lee, Alysa E. Doyle, Micah Silberstein, Jae-Yoon Jung, Richard Liu, Roy H. Perlis, Joshua Roffman, Jordan W. Smoller, Maurizio Fava, Ronald C. Kessler

**Affiliations:** 1 Center for Genomic Medicine, Massachusetts General Hospital, Boston; 2 Department of Psychiatry, Massachusetts General Hospital and Harvard Medical School, Boston; 3 Stanley Center for Psychiatric Research, Broad Institute of MIT and Harvard, Cambridge, Massachusetts; 4 Department of Pediatrics, Stanford University, Stanford, California; 5 Depression Clinical & Research Program, Massachusetts General Hospital, Boston; 6 Department of Health Care Policy, Harvard Medical School, Boston, Massachusetts

## Abstract

**Importance:**

Suicide rates have been increasing among youth in the US. While the heritability of suicide risk is well established, there is limited understanding of how genetic risk is associated with suicidal thoughts and behaviors in young children.

**Objective:**

To examine whether genetic susceptibility to suicide attempts (SAs) is associated with suicidal thoughts and behaviors in children.

**Design, Setting, and Participants:**

This case-control study examined data from the Adolescent Brain Cognitive Development (ABCD) study, a population-based longitudinal study of 11 878 US children enrolled at age 9 and 10 years from September 2016 to November 2018. Youth reports of suicidal ideation (SI) and SAs were obtained from the Kiddie Schedule for Affective Disorder and Schizophrenia at baseline and 2 subsequent years. After conservative quality control of genotype data, this analysis focused on 4344 unrelated individuals of European ancestry. Data analysis was conducted from November 2020 to February 2022.

**Main Outcomes and Measures:**

Children’s lifetime experiences of SI and SAs were assessed each year from ages 9 to 10 years to ages 11 to 12 years. Polygenic risk scores (PRSs) for SAs were calculated for ABCD study participants based on the largest genome-wide association study of SA cases and controls of European ancestry (total sample n = 518 612).

**Results:**

Of 4344 children of European ancestry (2045 [47.08%] female; mean [SD] age, 9.93 [0.62] years), significant associations were found between children’s SA PRSs and their lifetime SAs with the most robust association in the follow-up year 2 (odds ratio, 1.43 [95% CI, 1.18-1.75]; corrected *P* = 1.85 × 10^−3^; Nagelkerke pseudo* R^2^* = 1.51%). These associations remained significant after accounting for children’s sociodemographic backgrounds, psychopathology symptoms, parental histories of suicide and mental health, and PRSs for major depression and attention-deficit/hyperactivity disorder (likelihood ratio test *P* < .05). Children’s depressive mood and aggressive behavior were the most significant partial mediators of SA genetic risk on SAs (mediation analysis *P* < 1 × 10^−16^). Children’s behavioral problems, such as attention problems, rule-breaking behavior, and social problems, also partially mediated the association of SA PRSs with SAs (mediation analysis false discover rate < 0.05).

**Conclusions and Relevance:**

This study’s findings indicate that there may be genetic factors associated with SA risk across the life span and suggest behaviors and conditions through which the risk could be mediated in childhood. Further research is warranted to examine whether incorporating genetic data could improve the identification of children at risk for suicide.

## Key Points

**Question:**

Is genetic susceptibility to adulthood suicide attempts (SAs) associated with suicidal behaviors in children?

**Finding:**

In this case-control study of 4344 children using data from the population-based Adolescent Brain Cognitive Development (ABCD) study, statistically significant associations were found between children’s SAs and their polygenic risk scores for SAs, as derived from an independent genome-wide association study of 550 000 adults. These associations strengthened with age and remained significant after accounting for clinical, familial, and sociodemographic risk factors of suicide.

**Meaning:**

Genetic variation may influence the risk of SAs among children; risk stratification efforts, starting as early as preadolescence, may benefit from integrating genetic data.

## Teaser

This case-control study examines whether genetic susceptibility to suicide attempts is associated with suicidal thoughts and behaviors in children.

## Introduction

Suicide is a major public health problem worldwide, accounting for more than 1.3% of deaths each year.^1^ Despite active prevention efforts, death by suicide has increased steadily among US preadolescents since 2010.^2^ Suicidal thoughts and behaviors (STBs) are also common in young children, estimated at as high as 15% for suicidal ideation (SI) and 2.6% for suicide attempts (SAs).^2,3^ Yet, a lack of studies on risk factors for STBs in young children represents a major hurdle for early prevention and intervention efforts.^2,4^ Understanding the etiological bases of STBs will be critical for implementing evidence-based prevention strategies for children.^5,6^

The Adolescent Brain Cognitive Development (ABCD) study provides unprecedented opportunities to enhance our understanding of the etiologic basis of childhood suicide risk. This population-based longitudinal study of 11 878 US children enrolled participants during early elementary school (aged 9-10 years) with a plan to follow up with them for 10 years. The latest ABCD data released in 2021 (version 4.0) provides children’s suicide survey data collected on a yearly basis for 3 consecutive years, along with a wide range of phenotypic and genome-wide genetic data. To date, several studies of earlier ABCD data releases have reported distinct characteristics of children with STBs, including disadvantaged socioeconomic status, childhood psychopathology, and genetic associations with psychiatric disorders.^3,7,8,9,10,11^ In particular, our previous study^12^ using these data identified a significant association between childhood STBs and an increased burden of common genetic variants that confer risk for 2 psychiatric disorders, attention-deficit/hyperactivity disorder (ADHD) and major depressive disorder (MDD). Such findings support further research on genetic variation and suicide risk, which, in turn, may inform investigations of the utility of genetic information for suicide risk stratification in youth.

The primary aim of the present study was to further elucidate the genetic risk underlying suicide risk in children by capitalizing on newly available genome-wide association studies (GWASs) of SAs. Recent GWASs have established that SAs and suicide death are heritable, polygenic, and most importantly, share a genetic basis partially distinct from psychiatric disorders.^13,14,15,16,17,18^ However, understanding the relevance of these findings to risk mitigation efforts in youth requires closing current knowledge gaps. Important questions include (1) are polygenic risk scores (PRSs) for SAs associated with STBs in children? (2) If so, are the effects of SA PRSs independent of the genetic risk for psychiatric disorders, such as MDD and ADHD? Lastly, (3) do child temperaments and psychopathology problems mediate the effects of SA PRSs on children’s STBs? The present study aimed to address these questions by harnessing the power of the ABCD cohort and the latest GWAS data sets for SAs,^17^ each representing the largest genomics samples on STB outcomes in the field.

## Methods

### Study Cohort

The present study investigated ABCD data release version 4.0, downloaded from the National Institute of Mental Health Data Archive. The ABCD data included the full cohort data for the baseline and first 2 follow-up years, which was the focus of the present study. Children were enrolled from September 2016 to November 2018. Detailed information about sample collection, survey measures, and study protocols has been published elsewhere.^19,20,21^ Caregivers and the study participants provided informed consent for human research. The present study was approved by the Massachusetts General Hospital institutional review board. Additional information is provided in the eMethods in the Supplement.

### Outcome Measure

Using the youth report of the ABCD Kiddie Schedule for Affective Disorders and Schizophrenia for *DSM-5*, we generated cumulative lifetime measures for SI and SAs. In each year, participants were classified as SAs if they reported actual, interrupted, or aborted attempts at present or in the past. Participants were classified as SI when they reported SI but no SAs (at present or in the past). Controls were the ones with neither SI/SAs. Further details are described in eTable 1 in the Supplement.

### Genotyping, Data Quality Control, and Imputation

**Figure 1.**
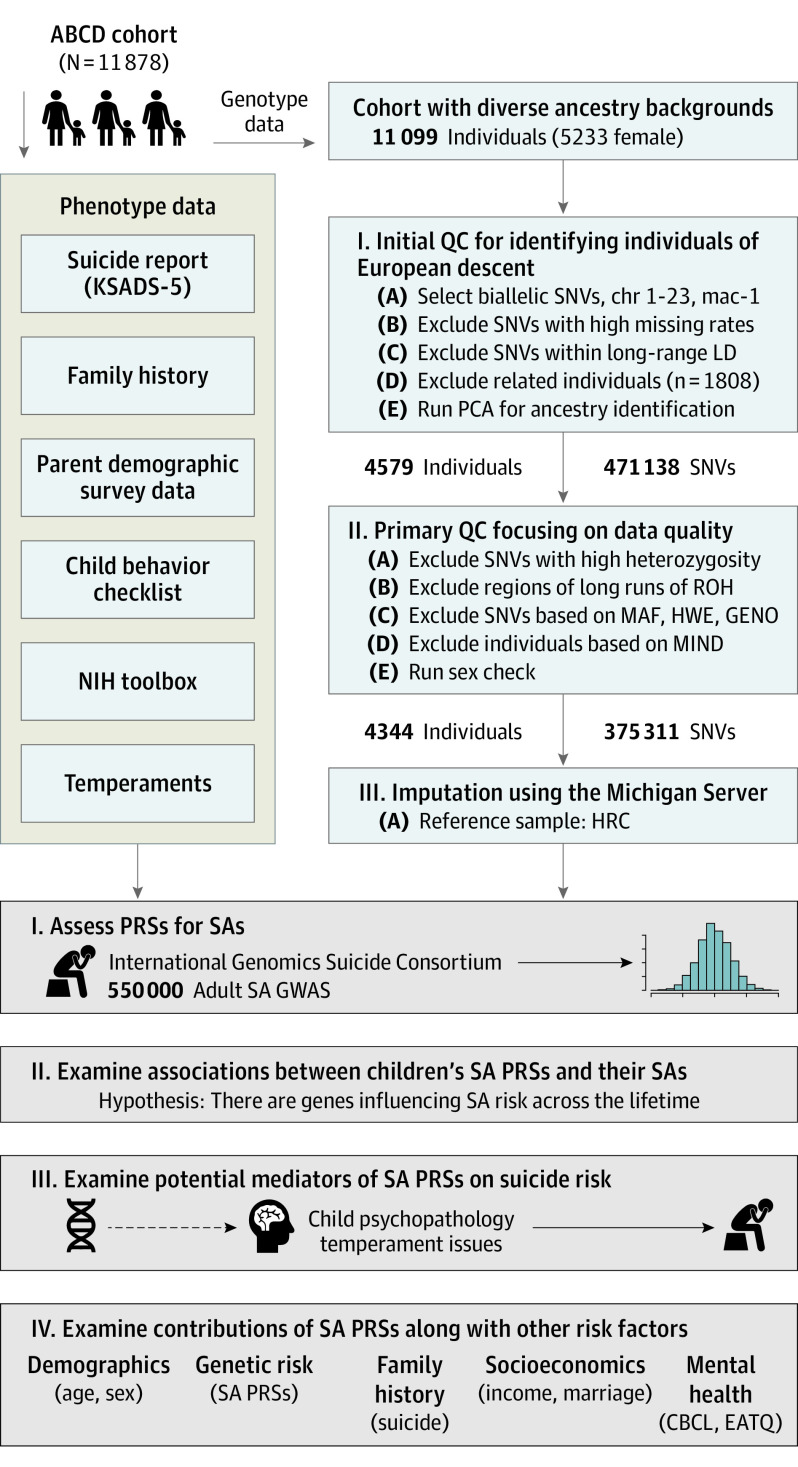
Outline of the Present Study Our study used Adolescent Brain Cognitive Development (ABCD) data release 4.0, which included genetics and phenotypic data collected at baseline and 2 follow-up years for 11 878 participants. Suicide risk outcomes were collected using the youth version of the computerized Kiddie Schedule for Affective Disorders and Schizophrenia for *DSM-5* (KSADS-5). Using the individual KSADS-5 item data, we generated cumulative suicide attempts (SA) and ideation measures in each year, starting from the baseline. For polygenic risk score (PRS) data generation, adult SA genome-wide association studies (GWAS) from the International Suicide Genomics Consortium was applied to 4344 ABCD participants of European ancestry. CBCL indicates Child Behavior Checklist; CHR, chromosome; EATQ, Early Adolescent Temperament Questionnaire; GENO, missing genotype rates; HRC, Haplotype Reference Consortium; HWE, Hardy-Weinberg Equilibrium; mac, minor allele count; MAF, minor allele frequency; MIND, missing genotype rates per individuals; NIH, National Institutes of Health; PCA, principal component analysis; QC, quality control; ROH, runs of homozygosity test; SNV, single-nucleotide variation.

Genome-wide genotype data, downloaded from the National Institute of Mental Health Data Archive, included 733 293 single-nucleotide variations (SNVs; formerly single-nucleotide polymorphisms or SNPs). Figure 1 summarizes the quality control procedures we applied to the genotype data. Principal component analysis with the 1000 Genomes Project reference samples identified 4344 individuals of European ancestry (eFigure 1 in the Supplement). Imputation was conducted using the Michigan Imputation Server (version 1.5.7) with minimac (version 4-1.0.2) and the Haplotype Reference Consortium panel. After excluding SNVs with imputation scores less than 0.8, a total of 6 960 459 SNVs were retained.

### PRS

To quantify children’s genetic susceptibility to SAs, we calculated PRSs, which represent the additive genome-wide genetic risk each individual carries. The risk alleles and effect size information for SAs were obtained from the latest International Suicide Genomics Consortium GWAS (26 590 cases and 492 022 controls of European ancestry).^17^ To date, this data set represents the largest, publicly available GWAS of SAs. PRS scores were generated using PRSice (version 2.3.3), with the 1K reference EUR samples. Default parameters were used for linkage-disequilibrium clumping and thresholds for SNV association *P *values were set to .10. Constructed SA PRSs showed a normal distribution (shapiro.test in R version 4.0.4 [R Foundation]; *P* > .05; eFigure 2 in the Supplement) and were not associated with demographic and technical confounders (eTable 2 in the Supplement).

### Demographic, Socioeconomic, Clinical, and Family History Data

We obtained the demographic information of the study participants, including age, sex, and socioeconomic status data from the ABCD Parent Demographics Survey. Children’s socioeconomic background data included parental marital status, poverty status, and parental college education. Child psychopathology data were obtained from the Child Behavior Checklist. Parental history of suicide and mental health data were obtained from the ABCD Parent Family History Summary Scores. For details of the instruments and variables, see eTable 3 in the Supplement.

### Statistical Analysis

Data analysis was conducted from November 2020 to February 2022. We examined the associations between children’s SA PRSs and their experiences of lifetime SAs and SI using multivariate logistic regression (R *glm* version 4.1 [R Foundation]). Each outcome measure was a binary dependent variable, while SA PRS was used as an independent predictor. Sex and age were used as covariates due to their associations with suicide risk outcomes. We also included top 10 genetic principal components as covariates to control for potential differences in population substructure within European individuals. All quantitative measures were scaled. The association of PRS with the outcome measure was estimated using the odds ratio (OR), which represents the likelihood of belonging to a suicide risk group when the PRS increases 1 SD from the norm. Bonferroni correction was used for multiple testing correction.

### Sensitivity Analysis

To check the robustness of the associations between SA PRSs and outcome measures, we examined whether significant findings remain consistent as the *P* value thresholds for constructing PRS change. We also examined whether SA PRSs show independent associations with outcome measures, while covarying with the PRSs for MDD and ADHD, 2 psychiatric disorders that we have previously identified significant associations with suicide risk in children. We also tested the associations of SA PRSs after adjusting for various clinical and familial risk factors for suicide (eTable 3 in the Supplement).

### Mediation Analysis

We examined potential mediation effect sizes of SA PRSs by various risk factors for suicide using the R package *mediation* version 3.0 (R Foundation). We examined 24 potential mediator variables, including 8 syndromic Child Behavior Checklist clinical problems and 10 personality/temperament measures based on the Early Adolescent Temperament Questionnaire (eTable 4 in the Supplement).

### Lasso Regression Analysis

We used the lasso regression package *glmnet* in R version 3.6 (R Foundation) to examine the independent associations between SA PRSs and children’s STBs while accounting for various suicide risk factors. Lasso regression minimizes the complexity of the model by applying shrinkage priors so that we can select an independent set of variables among potentially correlated variables. A binomial logistic model was fitted with a total of 30 variables. Once the set of predictors with nonzero effects were identified from lasso regression, we assessed the associations of the selected variables with children’s SAs using multivariate logistic regression. We provide detailed information of data sources, statistical/analytic tools, and method-specific references in eTable 5 in the Supplement.

## Results

### Prevalence and Characteristics of STBs in Children

The mean (SD) age of youth in our primary genetic data analysis (N = 4344 unrelated participants) was 9.93 (0.62) years at baseline (2045 [47.08%] female). The lifetime prevalence of SAs among the participants increased approximately 3 times from baseline to follow-up year 2 (baseline: 37 [0.85%]; year 1: 74 [1.7%]; year 2, 102 [2.35%]), while SI nearly doubled during the same period (baseline, 333 [7.67%]; year 1, 488 [11.23%]; year 2, 601 [13.84%]) (eTable 6 in the Supplement). Table 1 summarizes the main characteristics of these participants stratified by their suicide risk outcomes: SI, SAs, and the no SI/SA comparison group. Low socioeconomic status, parental psychiatric/suicide history, and child psychopathology were more common among children with SI and SAs compared with the control group. The follow-up years also showed similar findings (eTables 7 and 8 in the Supplement).

**Table 1.** Demographic, Socioeconomic, and Family History of the ABCD Participants Based on Youth-Reported Suicidal Behaviors at Baseline

Characteristic^a^	No. (%)			*P* value	
	SA (37 [0.85%])	SI (333 [7.67%])	Controls (3974 [91.48%])	SA vs controls	SI vs controls
Demographics					
Age, mean (SD), y	9.92 (0.63)	9.88 (0.61)	9.94 (0.62)	8.74 × 10^−1^	1.10 × 10^−1^
Female	16 (43.24)	138 (41.44)	1891 (47.58)	7.18 × 10^−1^	3.57 × 10^−2^^b^
Male	21 (56.76)	195 (58.56)	2083 (52.42)		
Socioeconomic status					
Poverty	6 (17.14)	15 (4.78)	147 (3.86)	3.60 × 10^−4^^b^	5.13 × 10^−1^
Single parents	17 (45.95)	81 (24.40)	675 (17.00)	9.92 × 10^−6^^b^	8.74 × 10^−4^^b^
No college degree	15 (40.54)	238 (71.47)	3061 (77.05)	4.81 × 10^−7^^b^	2.50 × 10^−2^^b^
Parental history					
Depression	11 (29.73)	50 (15.02)	360 (9.06)	5.47 × 10^−5^^b^	5.40 × 10^−4^^b^
Mental/emotional problems	6 (16.22)	28 (8.41)	176 (4.43)	2.43 × 10^−3^^b^	1.63 × 10^−3^^b^
Suicide	11 (29.73)	39 (11.71)	210 (5.28)	9.08 × 10^−10^^b^	2.54 × 10^−6^^b^
CBCL					
Anxious/depressed	9 (24.32)	24 (7.21)	100 (2.52)	2.68 × 10^−14^^b^	2.07 × 10^−6^^b^
Withdrawal/depressed	6 (16.22)	16 (4.80)	67 (1.69)	2.47 × 10^−9^^b^	1.64 × 10^−4^^b^
Somatic problems	6 (16.22)	15 (4.50)	100 (2.52)	3.22 × 10^−6^^b^	4.72 × 10^−2^
Social problems	6 (16.22)	10 (3.00)	43 (1.08)	3.21 × 10^−14^^b^	5.18 × 10^−3^^b^
Thought problems	11 (29.73)	29 (8.71)	157 (3.95)	1.59 × 10^−13^^b^	7.41 × 10^−5^^b^
Attention problems	7 (18.92)	17 (5.11)	101 (2.54)	1.96 × 10^−8^^b^	9.94 × 10^−3^^b^
Rule-breaking behavior	5 (13.51)	15 (4.50)	41 (1.03)	2.58 × 10^−10^^b^	3.03 × 10^−7^^b^
Aggressive behavior	7 (18.92)	13 (3.90)	50 (1.26)	7.65 × 10^−17^^b^	2.89 × 10^−4^^b^
Internal problems	12 (32.43)	26 (7.81)	110 (2.77)	1.91 × 10^−23^^b^	1.01 × 10^−6^^b^
External problems	6 (16.22)	13 (3.90)	51 (1.28)	3.89 × 10^−12^^b^	3.70 × 10^−4^^b^
Total problems	9 (24.32)	25 (7.51)	103 (2.59)	7.13 × 10^−14^^b^	9.29 × 10^−7^^b^
CBCL (*DSM-5*)					
Affective problems	9 (24.32)	32 (9.61)	87 (2.19)	1.90 × 10^−16^^b^	8.40 × 10^−15^^b^
Anxiety problems	8 (21.62)	22 (6.61)	111 (2.79)	4.60 × 10^−10^^b^	2.16 × 10^−4^^b^
Somatic problems	5 (13.51)	16 (4.80)	150 (3.77)	8.52 × 10^−3^^b^	4.30 × 10^−1^
Attention-deficit/hyperactivity disorder	5 (13.51)	15 (4.50)	100 (2.52)	2.59 × 10^−4^^b^	4.72 × 10^−2^
Opposite defiant problems	7 (18.92)	20 (6.01)	94 (2.37)	4.36 × 10^−9^^b^	1.46 × 10^−4^^b^
Conduct problems	6 (16.22)	13 (3.90)	62 (1.56)	4.55 × 10^−10^^b^	3.47 × 10^−3^^b^

Abbreviations: ABCD, Adolescent Brain Cognitive Development; CBCL, Child Behavior Checklist; SAs, suicide attempts; SI, suicidal ideation.

^a^ Demographic and family socioeconomic information was obtained from ABCD Parent Demographics Survey data. Lifetime SI and SA were generated based on 18-item Kiddie Schedule for Affective Disorders and Schizophrenia for *DSM-5* items that assess self-harm behaviors, passive or active thoughts of suicide, and SAs at present or in the past.

^b^ Significant statistical differences between suicide risk groups (ie, SA and SI) and controls after multiple testing correction are indicated (false discovery rate *q* ≤ 0.05).

### Association of Adult SA PRS and Suicide Risk in Children

**Figure 2.**
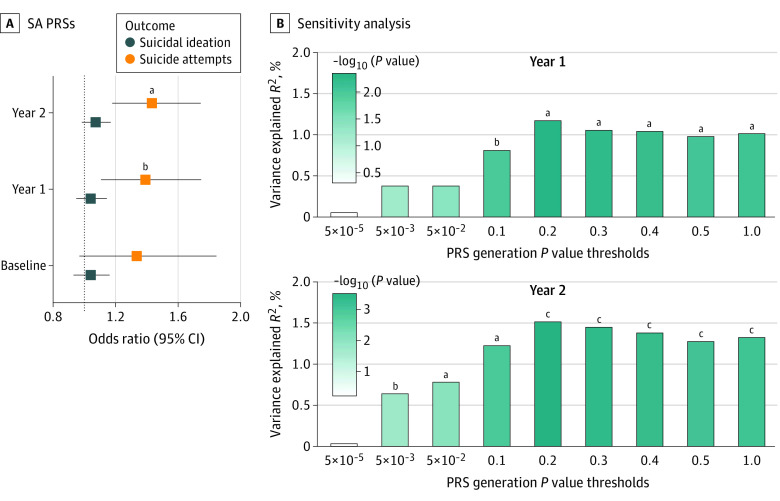
Polygenic Risk Score (PRS) Analysis Results on Children’s Suicidal Ideation and Attempt (SA) Outcomes A, Estimated odds ratios and 95% CIs of SA PRSs associated with children’s suicidal ideation and SAs. The dotted line represents a null effect (ie, odds ratio, 1.0). B, Sensitivity analysis results of SA PRSs associated with children’s SAs in the follow-up year 1 (top) and year 2 (bottom). The y-axis represents the percentage of total variance explained by SA PRS (Nagelkerke pseudo *R*^2^), controlling for age, sex, and top 10 principal components covariates. The x-axis represents 10 *P* value thresholds used to generate SA PRSs. The *P* value significance of the estimated β coefficients of each PRS is displayed in the top of the bars. Distinct colors were used to represent the statistical significance of the PRS effect size. ^a^.001 < *P* ≤ .01. ^b^*P* ≤ 1 × 10^−3^. ^c^.01 < *P* ≤ .05.

We examined the associations between children’s genetic liability to SAs, quantified as genome-wide SA PRSs, and their lifetime experiences of SI and SAs. Effect sizes of SA PRSs on children’s SAs (ie, ORs) ranged from 1.34 to 1.43 (Figure 2A and eTable 9 in the Supplement). As the number of children with SAs increased each year, the association of SA PRSs with youth SAs became more pronounced and remained statistically significant after multiple testing correction in year 1 (OR, 1.39 [95% CI, 1.11-1.75]; corrected *P* = 2.73 × 10^−2^) and year 2 (OR, 1.43 [95% CI, 1.18-1.75]; corrected *P* = 1.85 × 10^−3^). In contrast, the association between SA PRSs and SI was not significant at any time period (ORs range, 1.04-1.07 [95% CI, 0.93-1.17]). Sensitivity analysis confirmed that the associations of SA PRSs with children’s SAs remained robust across a range of conditions (Figure 2B and eTable 10 and eFigures 3-5 in the Supplement). Similar to our primary analyses, we did not find associations between additional *P* value thresholds of SA PRSs with SI (eFigure 6 and eTable 11 in the Supplement).

### Association of SA PRSs With Genetic Risk of Psychiatric Disorders

Next, we examined whether the associations identified between children’s SA PRSs and SAs were independent from the genetic influences of MDD and ADHD. In line with our previous study,^12^ PRSs for these 2 psychiatric disorders were significantly associated with children’s SAs and SI, respectively (eTable 12 in the Supplement). Nevertheless, the associations between SA PRSs and youth SAs observed at years 1 and 2 remained significant after covarying for MDD and ADHD PRSs (likelihood ratio test *P* < .05; eTable 13 in the Supplement). Nagelkerke *R*^2^ indicated that SA PRSs additionally explained 0.43% to 1.18% of variance in children’s SAs, independent of MDD and ADHD PRSs.

### Mediating Association of SA PRS With Known Risk Factors of Suicide

We then examined whether the associations identified between SA PRSs and children’s SAs were mediated by known risk factors of suicide. Specifically, we hypothesized that genetic risk underlying SAs may increase children’s STBs through child psychopathology (eg, depression, anxiety) or temperament issues (eg, aggression, impulsivity). Table 2 summarizes the mediation analysis results (full data in eTable 14 in the Supplement). Of the 8 Child Behavior Checklist measures we examined, statistically significant mediation effect sizes were found between SA PRSs and 5 psychopathology problems on SAs, most notably, aggressive behaviors (mediation analysis mediation effect *P *<* *2* × *10^−16^; full data are available in Table 2 and eTable 14 in the Supplement). Among 10 temperament measures, we found the most significant mediation effect sizes of SA PRSs on depressive mood (mediation analysis: OR, 2.46 [95% CI, 2.01-3.01]; *P *<* *2* × *10^−16^). However, in all analyses nonmediated, direct effect sizes of SA PRSs on SAs remained statistically significant.

**Table 2.** Mediation Analysis Results Between SA PRSs, Mediator Variables, and Children's SA Outcome^a^

Mediator	SA PRS on mediator		Mediator on SA		Mediation effects			
	OR (95% CI)	*P* value	OR (95% CI)	*P* value	MP (95% CI)	*P* value		FDR (ACME)
						ADE	ACME	
Psychopathology (CBCL syndrome)								
Aggressive behavior	1.06 (1.03 to 1.10)	4.36 × 10^−4^^b^	1.72 (1.51 to 1.96)	3.54 × 10^−16^^b^	0.11 (0.04 to 0.43)	.004	<2 × 10^−16^	<2e × 10^−16^^c^
Anxious depressed	1.03 (1.00 to 1.07)	6.67 × 10^−2^	1.94 (1.68 to 2.23)	<2 × 10^−16^^b^	0.06 (0 to 0.22)	.004	.056	1.24 × 10^−1^
Attention problems	1.06 (1.02 to 1.10)	9.58 × 10^−4^^b^	1.66 (1.44 to 1.91)	2.33 × 10^−12^^b^	0.09 (0.03 to 0.40)	.02	.004	1.55 × 10^−2^^c^
Rule-breaking behavior	1.05 (1.02 to 1.09)	2.91 × 10^−3^^d^	1.76 (1.54 to 2.01)	<2 × 10^−16^^b^	0.09 (0.03 to 0.43)	.02	.004	1.55 × 10^−2^^c^
Social problems	1.05 (1.01 to 1.09)	5.27 × 10^−3^^d^	1.82 (1.59 to 2.08)	<2 × 10^−16^^b^	0.09 (0.02 to 0.39)	.004	.008	2.25 × 10^−2^^c^
Somatic complaints	1.04 (1.01 to 1.08)	1.57 × 10^−2^^e^	1.63 (1.36 to 1.96)	1.29 × 10^−7^^b^	0.06 (0.01 to 0.18)	.008	.008	2.25 × 10^−2^^c^
Thought problems	1.03 (1.00 to 1.07)	7.61 × 10^−2^	2 (1.70 to 2.35)	<2 × 10^−16^^b^	0.06 (0 to 0.25)	<2 × 10^−16^	.06	1.24 × 10^−1^
Withdrawn depressed	1.03 (0.99 to 1.06)	1.46 × 10^1^	1.72 (1.49 to 1.99)	1.74 × 10^−13^^b^	0.04 (−0.02 to 0.19)	.004	.14	2.22 × 10^−1^
Temperament (EATQ-R)								
Activation control	1.01 (0.98 to 1.05)	3.88 × 10^−1^	0.87 (0.71 to 1.06)	1.64 × 10^−1^	−0.01 (−0.04 to 0.01)	<2 × 10^−16^	.50	6.15 × 10^−1^
Affiliation	0.99 (0.96 to 1.02)	5.44 × 10^−1^	0.74 (0.61 to 0.90)	2.91 × 10^−3^^d^	0.01 (−0.02 to 0.05)	<2 × 10^−16^	.48	6.15 × 10^−1^
Aggression	1.04 (1.01 to 1.07)	1.08 × 10^−2^^e^	1.88 (1.55 to 2.28)	1.43 × 10^−10^^b^	0.08 (0.02 to 0.26)	.004	.008	2.13 × 10^−2^^c^
Attention	1.0 (0.97 to 1.03)	8.81 × 10^−1^	0.88 (0.72 to 1.08)	2.16 × 10^−1^	0 (−0.02 to 0.04)	.008	.93	9.28 × 10^−1^
Depressive mood	1.04 (1.01 to 1.07)	1.05 × 10^−2^^e^	2.46 (2.01 to 3.01)	<2 × 10^−16^^b^	0.12 (0.03 to 0.47)	.02	<2 × 10^−16^	<2 × 10^−16^^c^
Fear	1.03 (1.00 to 1.06)	7.27 × 10^−2^	1.71 (1.40 to 2.10)	2.16 × 10^−7^^b^	0.05 (−0.01 to 0.16)	.004	.06	1.20 × 10^−1^
Frustration	1.03 (1.00 to 1.07)	3.27 × 10^−2^^e^	1.84 (1.49 to 2.29)	2.81 × 10^−8^^b^	0.06 (0.00 to 0.17)	.004	.048	1.10 × 10^−1^
High-intensity pleasure/surgency	1.01 (0.98 to 1.04)	6.91 × 10^−1^	0.69 (0.56 to 0.84)	2.35 × 10^−4^^b^	−0.01 (−0.06 to 0.03)	.004	.71	8.09 × 10^−1^
Inhibitory control	1.02 (0.99 to 1.05)	1.78 × 10^−1^	1.39 (1.13 to 1.71)	1.56 × 10^−3^^d^	0.02 (−0.01 to 0.09)	<2 × 10^−16^	.19	2.76 × 10^−1^
Shyness	1 (0.96 to 1.03)	7.76 × 10^−1^	0.84 (0.69 to 1.02)	7.34 × 10^−2^	0 (−0.02 to 0.03)	<2e × 10^−16^	.86	9.17 × 10^−1^

Abbreviations: ACME, average causal mediation effect; ADE, average direct effect; CBCL, Child Behavior Checklist; EATQ-R, Early Adolescent Temperament Questionnaire Revised; FDR, false discovery rate; MP, mediated proportion; OR, odds ratio; PRSs, polygenic risk scores; SAs, suicide attempts.

^a^ In the mediation analysis, multivariable logistic regression was used where SA PRSs and each mediator were used as independent variables for predicting the SA outcome. In all regression, age, sex, and top 10 genetic principal components were used as covariates.

^b^ *P <* .001.

^c^ Significant mediation and direct effects after multiple testing correction are indicated (false discovery rate *q* ≤ 0.05).

^d^ .001 <* P <* .001.

^e^ .01 <* P <* .05.

### Independent Contributions of Suicide Risk Factors

Last, we applied lasso regression to test the independent contribution of SA PRSs on children’s SA risk along with other suicide risk variables (see Methods). Table 3 summarizes the analysis results for year 2 (further data in eTable 15 and eFigure 7 in the Supplement). Of the 30 variables we examined in the same model, children’s socioeconomic backgrounds, specifically single-parent status (OR, 2.2 [95% CI, 1.24-3.88]; *P* = 6.74* × *10^−3^) and a lack of parents’ college education (OR, 1.78 [95% CI, 1.01-3.15]; *P* = 4.66* × *10^−2^) were the most significant correlates of children’s SAs. Children’s depressive mood (OR, 1.47 [95% CI, 1.1-1.96]; *P* = 8.26* × *10^−3^) and reduced pleasure in activities in high intensity or novelty (OR, 1.41 [95% CI, 1.09-1.81]; *P* = 7.57* × *10^−3^) were ranked next. Despite the modest OR, we confirmed that SA PRSs contributed independently to the prediction model (OR, 1.4 [95% CI, 1.08-1.82]; *P* = 1.07* × *10^−2^; Nagelkerke *R*^2^ = 1.39%).

**Table 3.** Lasso Logistic Regression Analysis Results of 30 Variables and Children’s SAs^a^

Factor	Lasso regression coefficient	Multivariable logistic regression	
		OR (95% CI)	*P* value
Demographic			
Age	NA^b^	NA	NA
Sex	NA^b^	NA	NA
Genetic risk			
MDD PRS	0.152	1.34 (1.01-1.78)	4.10 × 10^−2^^c^
ADHD PRS	NA	NA	NA
SA PRS	0.212	1.4 (1.08-1.82)	1.07 × 10^−2^^c^
Socioeconomic status			
Single parents	0.591	2.2 (1.24-3.88)	6.74 × 10^−3^^d^
Parents' lack of college education	0.414	1.78 (1.01-3.15)	4.66 × 10^−2^^c^
Poverty	NA^b^	NA	NA
Household income	NA^b^	NA	NA
Parental history			
Mental problems	0.009	1.25 (0.54-2.91)	6.03 × 10^−1^
Depression	NA	NA	NA
Suicide	0.244	1.31 (0.61-2.83)	4.89 × 10^−1^
Child Psychopathology (CBCL syndromes)			
Anxious depression	0.264	1.3 (1.04-1.63)	2.40 × 10^−2^^c^
Withdrawal depression	NA^b^	NA	NA
Somatic complaints	NA^b^	NA	NA
Social problems	0.128	1.13 (0.93-1.38)	2.18 × 10^−1^
Thought problems	NA^b^	NA	NA
Attention problems	NA^b^	NA	NA
Rule-breaking behaviors	0.073	1.05 (0.83-1.33)	6.62 × 10^−1^
Aggressive behaviors	0.162	1.2 (0.93-1.55)	1.52 × 10^−1^
Child temperament (EATQ)			
Activation control	NA^b^	NA	NA
Affiliation	NA^b^	NA	NA
Attention	NA^b^	NA	NA
Fear	NA^b^	NA	NA
Frustration	NA^b^	NA	NA
High-intensity pleasure/surgency	0.174	1.41 (1.09-1.81)	7.57 × 10^−3^^d^
Inhibitory control	0.007	1.16 (0.89-1.51)	2.77 × 10^−1^
Shyness	NA^b^	NA	NA
Aggression	NA^b^	NA	NA
Depressive mood	0.327	1.47 (1.1-1.96)	8.26 × 10^−3^^d^

Abbreviations: ADHD, attention-deficit/hyperactivity disorder; CBCL, Child Behavior Checklist; EATQ, Early Adolescent Temperament Questionnaire; MDD, major depressive disorder; NA, not applicable; OR, odds ratio; PRSs, polygenic risk scores; SAs, suicide attempts.

^a^ Lasso regression estimated the coefficient of each variable, while excluding less important features in the model. For the variables with nonzero coefficients, we applied multivariable logistic regression analysis to measure the relationship of each variable with suicide attempts. To estimate the independent association of SA PRSs, SA PRSs were used to estimate the selected variables and the residuals were used in the subsequent multivariate logistic regression. This guaranteed all variables to be uncorrelated with SA PRSs and dissociate the association of SA PRSs from others. The results are displayed for each independent variable used in the model using: odds ratio (the exponential of the logistic regression β coefficient), standard error of regression estimates, and *P* value (significance of estimated β using z statistic).

^b^ Less important features in the model.

^c^ .01 < *P* ≤ .05.

^d^ .001 < *P* ≤ .01.

## Discussion

Despite the alarming rate of suicide in youth, there have been limited empirical investigations of risk factors for STBs in younger children.^2^ In this study, we examined the genetic correlates of children’s STBs using the largest US sample of 4344 elementary school–aged children. Our study provides, to our knowledge, the first empirical evidence that common genetic variants associated with the increased risk of SAs are associated with SAs in children. These associations of SA PRS with child SAs were independent of genetic risk for MDD and ADHD and significant even after accounting for clinical, sociodemographic, and family risk factors of suicide.

Our study advances the field of suicide research in young children in several important ways. First, our results extend prior epidemiological evidence^22^ that suicide risk in this age group is relevant to genetic influences on SA risk that manifest across the life span and may begin in childhood. The genetic association of adult SA risk was detected in children as early as age 10 and 11 years and became more robust through the subsequent year. Although not previously demonstrated in molecular genetics studies, this finding is in line with the documented familial transmission of suicide,^22,23^ including evidence linking suicide in parents to SAs and death by suicide in their youth offspring.^24,25,26^

Second, our data extend evidence that the genetic risk for SAs is at least in part independent of the risk for psychiatric illness. Although there is strong evidence that psychiatric conditions, particularly MDD, are risk factors for suicide,^27,28^ data from twin and family studies have suggested an independent heritable component for SAs.^29,30^ The source GWAS for our SA PRSs^17^ corroborated this possibility by showing that the SNV-based heritability *h^2^* for SAs remained significant, although reduced, after conditioning on MDD. In the current study, we provide additional evidence for this distinction, as the association between children’s SAs and SA PRSs remained significant along with the significant associations with MDD PRSs (eTable 16 in the Supplement for further analysis results). Intriguingly, the pattern of findings differed for SI in our study. Specifically, ADHD PRSs (but none of SA or MDD PRSs) showed significant associations with SI. This result echoes family data^29^ in suggesting that the genetic risk for SAs that is distinguishable from the risk for psychiatric illness may be more strongly related to SAs than SI.

Third, our mediation analysis provides novel insights into how genetic risk may manifest in relation to children’s STBs. A significant proportion of SA genetic risk was mediated through children’s depressive mood and aggressive behaviors. Prior data suggest that SAs occur at younger ages in individuals with impulsive aggression.^31^ Indeed, King and colleagues^32^ identified impulsive aggression as a distinct risk profile, along with depression, in conferring risk for SAs in youth. While there is mixed evidence regarding the role of aggression or irritability in predicting SAs,^28,32,33,34,35,36,37^ our results support further investigation into the possibility that targeting aggressive behaviors, in addition to depression, could mitigate the impact of genetic risk for SAs in children.

Lastly, our results support further investigation of the potential role for genomic information^38^ in the early identification of children at risk for suicide. We observed a roughly 3-fold increase in the prevalence of SAs in children during the 3-year period we examined. With more variation in the outcome, the strength of the association with SA genetic risk also increased. The amount of phenotypic variance explained by SA PRSs in children was also somewhat larger compared with previous PRS studies of adults.^16,17,18,39^ In the article by Mullins et al,^17^ SA PRSs explained 0.68% to 0.88% of SA phenotypic variance in adult psychiatric patients and 1.08% of death by suicide in the Utah cohort.^18^ In the study by Campos et al,^39^ self-harm PRS explained 0.21% of SAs in UK Biobank participants. Of course, SA PRS explained at most 1.51% of the variance in childhood SAs in our analyses and would not have clinical utility on their own. The published literature and our own data also highlights phenotypic, socioeconomic, and psychosocial factors that are stronger predictors of suicide risk in youth.^27^ Additionally, ethical concerns for using genetic data for individual-level prediction of suicide risk should be adequately addressed.^40^ Nonetheless, given the potential for PRS to contribute information prior to the emergence of SAs and to serve as objective indicators where no biomarkers currently exist, our data suggest that it may be reasonable to study genetic information along with other risk factors for improving suicide risk stratification and prevention efforts in children.

### Limitations

Our study should be interpreted in light of several limitations. First, the sample studied here represent a population-based cohort, enrolled at age 9 and 10 years and followed up for 2 successive years. Further work is needed to determine the extent to which our findings generalize to different age groups or to clinically referred youth. Second, our analyses were limited to the participants of European ancestry as the discovery SA GWAS was based on exclusively European ancestry samples and the relatively small sample sizes of non-European samples (eTable 17 in the Supplement). It is critical that GWAS and other pediatric studies examining risk indicators and etiologic mechanisms be extended to Black and Latinx youth, whose high risk for suicide is increasingly evident^41,42,43^ and who may experience structural inequities that create additional risk factors for this outcome. Third, we acknowledge that the effect size for our SA PRSs were of small magnitude and do not resolve the challenge of individual-level prediction of SA.^27^

## Conclusions

Despite these limitations, our findings substantially advance the sparse empirical literature on suicide genetics research in children. Using newly available GWAS for SAs in relation to the richly phenotyped population cohort of US youth with genome-wide genetic data, our study demonstrates robust associations between genetic risk for SAs and SAs in young children and potential mediators of this genetic risk. Considering the increasing rates of STBs in youth, as observed in the ABCD cohort, further research is warranted to dissect the complex interplay of genetic and environmental risk factors and to set the stage for improved suicide prevention and intervention efforts.

## Notes

Supplement. eMethods. eTable 1. Details of K-SADS-5 variables used for constructing lifetime suicide risk outcome measures eTable 2. Correlations between SA PRSs and key demographic, andromorphic, ABCD study sites, and top ten genetic principal components (PC) eTable 3. Summary of ABCD variables used as predictors in multiple logistic regression for suicide risk outcome eTable 4. Summary of ABCD variables tested for potential mediators in causal mediation analysis eTable 5. List of data and methods used in the present study eTable 6. Prevalence of children with suicide attempts (SAs), suicidal ideation (SI), and no SA/SI experiences over three consecutive years eTable 7. Distinct characteristics of children with suicide attempts (SAs), suicidal ideation (SI), and no SA/SI experiences in Year 1 eTable 8. Distinct characteristics of children with suicide attempts (SAs), suicidal ideation (SI), and no SA/SI experiences in Year 2 eTable 9. Results of logistic regression analysis for suicide attempts (SA) polygenic risk scores (PRSs) on children’s lifetime suicide risk outcome measures eTable 10. Sensitivity analysis results of suicide attempts (SA) polygenic risk scores (PRSs) on SA risk model with respect to different p-value thresholds eTable 11. Sensitivity analysis results of suicide attempts (SA) polygenic risk scores (PRSs) on suicidal ideation (SI) risk model with respect to different p-value thresholds eTable 12. Results of logistic regression analysis for major depression (MD) and attention deficit/hyperactivity disorder (ADHD) polygenic risk scores (PRSs) on predicting children’s lifetime suicide risk outcome measures eTable 13. Logistic regression analysis results of suicide attempts (SA) polygenic risk scores (PRSs) on children’s lifetime SA outcome measures, conditioning on major depression (MD) and attention deficit/hyperactivity disorder (ADHD) PRSs eTable 14. Mediation analysis results between SA PRSs, mediator variables, and SA eTable 15. Univariate logistic regression analysis results of 30 predictors on SA outcome eTable 16. Additional analysis of cross-disorder GWAS data eTable 17. Logistic regression analysis of non-European sample analysis eFigure 1. Genetic diversity of ABCD participants eFigure 2. Characteristics of polygenic risk scores (PRSs) of adulthood suicide attempts (SAs) eFigure 3. Violin plots for SA PRSs stratified by the status of lifetime SAs from the baseline to year 2 eFigure 4. Beeswarm plot of SA PRSs in Year 2 eFigure 5. Beeswarm plot of SA PRSs in Year 2, with the same number of SA vs. no SA/SI participants eFigure 6. Sensitivity analysis results of children’s suicide attempt (SA) polygenic risk scores (PRSs) and their lifetime suicidal ideation eFigure 7. Correlations between 30 predictors used in lasso regression eReferences
