# Supplementary material for: Associations Between Genetic Risk for Adult Suicide Attempt and Suicidal Behaviors in Young Children in the US
Source: JAMA Psychiatry. 2022 Aug 31;79(10):971–80. doi: 10.1001/jamapsychiatry.2022.2379 (PMC9434482; doi:10.1001/jamapsychiatry.2022.2379)
Supplement: Supplement. — eMethods. eTable 1. Details of K-SADS-5 variables used for constructing lifetime suicide risk outcome measures eTable 2. Correlations between SA PRSs and key demographic, andromorphic, ABCD study sites, and top ten genetic principal components (PC) eTable 3. Summary of ABCD variables used as predictors in multiple logistic regression for suicide risk outcome eTable 4. Summary of ABCD variables tested for potential mediators in causal mediation analysis eTable 5. List of data and methods used in the present study eTable 6. Prevalence of children with suicide attempts (SAs), suicidal ideation (SI), and no SA/SI experiences over three consecutive years eTable 7. Distinct characteristics of children with suicide attempts (SAs), suicidal ideation (SI), and no SA/SI experiences in Year 1 eTable 8. Distinct characteristics of children with suicide attempts (SAs), suicidal ideation (SI), and no SA/SI experiences in Year 2 eTable 9. Results of logistic regression analysis for suicide attempts (SA) polygenic risk scores (PRSs) on children’s lifetime suicide risk outcome measures eTable 10. Sensitivity analysis results of suicide attempts (SA) polygenic risk scores (PRSs) on SA risk model with respect to different p-value thresholds eTable 11. Sensitivity analysis results of suicide attempts (SA) polygenic risk scores (PRSs) on suicidal ideation (SI) risk model with respect to different p-value thresholds eTable 12. Results of logistic regression analysis for major depression (MD) and attention deficit/hyperactivity disorder (ADHD) polygenic risk scores (PRSs) on predicting children’s lifetime suicide risk outcome measures eTable 13. Logistic regression analysis results of suicide attempts (SA) polygenic risk scores (PRSs) on children’s lifetime SA outcome measures, conditioning on major depression (MD) and attention deficit/hyperactivity disorder (ADHD) PRSs eTable 14. Mediation analysis results between SA PRSs, mediator variables, and SA eTable 15. Univariate logistic regres [file jamapsychiatry-e222379-s001.pdf]

Supplemental Online Content

Lee PH, Doyle AE, Silberstein M, et al. Associations between genetic risk for adult suicide attempt and suicidal behaviors in young children in the US. *JAMA Psychiatry*. Published online August 31, 2022. doi:10.1001/jamapsychiatry.2022.2379

eMethods.
eTable 1. Details of K-SADS-5 variables used for constructing lifetime suicide risk outcome measures
eTable 2. Correlations between SA PRSs and key demographic, andromorphic, ABCD study sites, and top ten genetic principal components (PC)
eTable 3. Summary of ABCD variables used as predictors in multiple logistic regression for suicide risk outcome
eTable 4. Summary of ABCD variables tested for potential mediators in causal mediation analysis
eTable 5. List of data and methods used in the present study
eTable 6. Prevalence of children with suicide attempts (SAs), suicidal ideation (SI), and no SA/SI experiences over three consecutive years
eTable 7. Distinct characteristics of children with suicide attempts (SAs), suicidal ideation (SI), and no SA/SI experiences in Year 1
eTable 8. Distinct characteristics of children with suicide attempts (SAs), suicidal ideation (SI), and no SA/SI experiences in Year 2
eTable 9. Results of logistic regression analysis for suicide attempts (SA) polygenic risk scores (PRSs) on children's lifetime suicide risk outcome measures
eTable 10. Sensitivity analysis results of suicide attempts (SA) polygenic risk scores (PRSs) on SA risk model with respect to different p-value thresholds
eTable 11. Sensitivity analysis results of suicide attempts (SA) polygenic risk scores (PRSs) on suicidal ideation (SI) risk model with respect to different p-value thresholds
eTable 12. Results of logistic regression analysis for major depression (MD) and attention deficit/hyperactivity disorder (ADHD) polygenic risk scores (PRSs) on predicting children's lifetime suicide risk outcome measures
eTable 13. Logistic regression analysis results of suicide attempts (SA) polygenic risk scores (PRSs) on children's lifetime SA outcome measures, conditioning on major depression (MD) and attention deficit/hyperactivity disorder (ADHD) PRSs
eTable 14. Mediation analysis results between SA PRSs, mediator variables, and SA
eTable 15. Univariate logistic regression analysis results of 30 predictors on SA outcome
eTable 16. Additional analysis of cross-disorder GWAS data
eTable 17. Logistic regression analysis of non-European sample analysis
eFigure 1. Genetic diversity of ABCD participants
eFigure 2. Characteristics of polygenic risk scores (PRSs) of adulthood suicide attempts (SAs)

© 2022 Lee et al. *JAMA Psychiatry.*

eFigure 3. Violin plots for SA PRSs stratified by the status of lifetime SAs from the baseline to year 2
eFigure 4. Beeswarm plot of SA PRSs in Year 2
eFigure 5. Beeswarm plot of SA PRSs in Year 2, with the same number of SA vs. no SA/SI participants
eFigure 6. Sensitivity analysis results of children's suicide attempt (SA) polygenic risk scores (PRSs) and their lifetime suicidal ideation
eFigure 7. Correlations between 30 predictors used in lasso regression
eReferences

This supplemental material has been provided by the authors to give readers additional information about their work.

© 2022 Lee et al. *JAMA Psychiatry*.

eMethods

Study Cohort
The present study investigated ABCD Data Release v4.0, downloaded from the National Institute of Mental Health Data Archive (NDA) (https:// data-archive.nimh.nih.gov/abcd). The ABCD data release (v4.0) included the full cohort data for the baseline and first two follow-up years, which was the focus of the present study. Detailed information about sample collection, survey measures, and study protocols has been published elsewhere[1-3]. Caregivers and the study participants of the ABCD study provided informed consent for human research. The present study was approved by the Massachusetts General Hospital IRB (Boston, US) as a secondary analysis of publicly available ABCD datasets (#2021P001872).

Outcome Measure
The ABCD computerized Kiddie Schedule for Affective Disorders and Schizophrenia for Diagnostic and Statistical Manual for Mental Disorders (K-SADS-PL DSM-5)[4] included a suicide module that asks study participants about their past and present experiences of active and passive SI as well as aborted and interrupted SAs. The survey was completed by the study participants on a yearly basis, starting from the baseline. Using the youth-report data, we generated two aggregated lifetime measures for SI and SAs in each year. When a participant reported both SI and SAs, we classified the person as SA. Thus, our three outcomes were SI, SAs (with or without SI), and controls with neither SI/SAs.

Exposure
To quantify children's genetic susceptibility to SAs, we calculated polygenic risk scores (PRSs), which represent the additive genome-wide genetic risk individuals carry based on the risk alleles and effect size information obtained from independent GWAS datasets. Specifically, we used the latest genome-wide summary statistics data for SAs obtained from the International Suicide Genomics Consortium (ISGC) (N=29,782 cases and 519,961 controls of European ancestry)[5]. All study participants in the ISGC GWAS were adults of minimum 19 years old. To date, this dataset represents the largest, publicly available GWASs of adult SAs. Genetic variants with independent associations with SA were identified using PLINK clumping (linkage-disequilibrium r^2=0.8, association p-value < 0.05) and were scored using PRSice (v2.3.3). Constructed SA PRSs of the ABCD children followed a normal distribution (shapiro.test in R p-value>0.05) and were not associated with demographic and technical confounders.

Sensitivity Analysis
To check the robustness of the associations between SA PRSs and outcome measures, we examined how the regression results change across 10 p-value thresholds: $5x10^{-5}$, $5x10^{-4}$, $5x10^{-3}$, $5x10^{-2}$, 0.1, 0.2, 0.3, 0.4, 0.5, and 1.0. We also examined whether SA PRSs show independent associations with outcome measures, while covarying with the PRSs for MDD and ADHD, two psychiatric disorders that we have previously identified as significantly associated with suicide risk in children. We also tested the associations of SA PRSs after adjusting for various risk factors for suicide, summarized in **eTable 3**.

Lasso Regression Analysis
We used the lasso regression package *glmnet* in R (v3.6) to examine the independent contributions of SA PRSs on predicting children's SBs while accounting for various suicide risk factors. Lasso regression minimizes the complexity of the model by applying shrinkage priors so that we can select an independent set of predictors among potentially correlated variables. A binomial logistic model was fitted with a total of 30 predictors. The risk predictors belonged to 6 categories: (1) demographics (age, sex); (2) socioeconomic status (parents' marriage status, parents' college education, household income, and poverty); (3) family history (parents' mental problems, depression, and suicide); (4) child psychopathology (8 CBCL syndromic problem T-scores); (5) child temperament (10 EATQ measures) and (6) child genetic risk (MDD, ADHD, and SA PRSs). The penalty parameter *alpha* was set to 1 with default settings for others. Once the set of predictors with non-zero effects were identified from lasso regression, we assess the effects of the selected predictors on children's SAs using multivariable logistic regression. Residualization of SA PRSs was conducted to separate its individual effect from other predictors[6].

© 2022 Lee et al. *JAMA Psychiatry.*

eFigure 1. Genetic diversity of ABCD participants. The first three principal components of ABCD genetic samples are displayed in the X and the Y-axis respectively. Each dot on the plot represents individuals. ABCD participants who clustered with the 1000 Genomes Project European references are highlighted in red among other ABCD participants in gray.

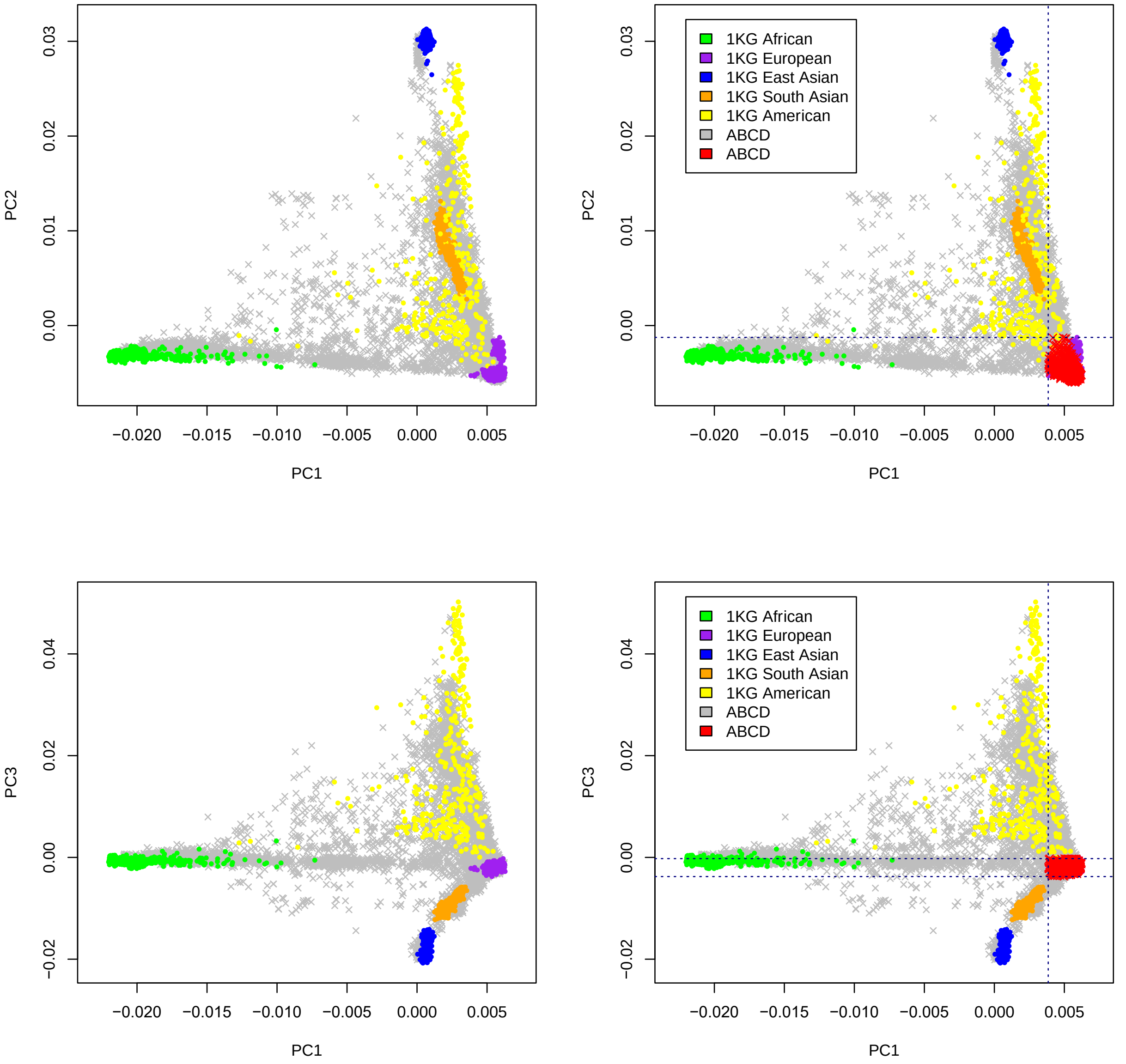

© 2022 Lee et al. *JAMA Psychiatry*.

eFigure 2. Characteristics of polygenic risk scores (PRSs) of adulthood suicide attempts (SAs). Normality of PRSs was confirmed using Shapiro's test in R, consistent with visualization results of three distribution plots (based on frequency, density, and quantiles). For PRS analysis, standardization was applied as displayed in the last plot.

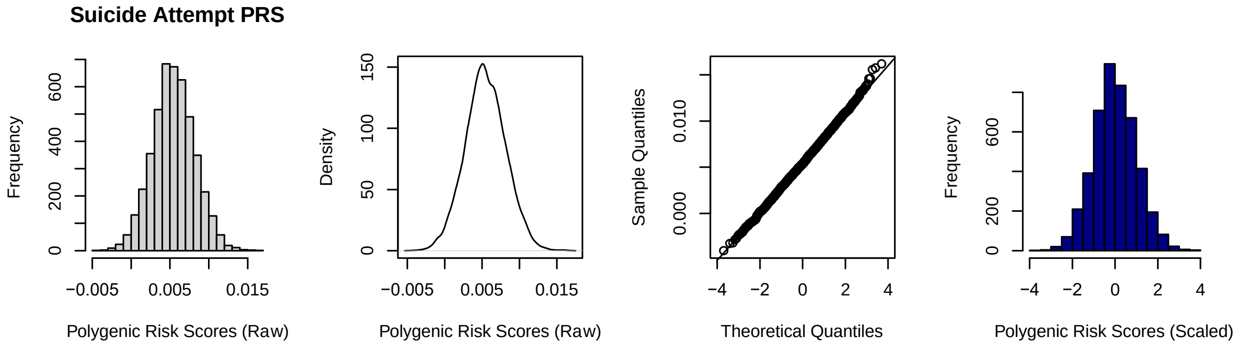

© 2022 Lee et al. *JAMA Psychiatry*.

eFigure 3. Violin plots for SA PRSs stratified by the status of lifetime SAs from the baseline to year 2. Y-axis represents SA PRSs, which were regressed out for top ten genetic components and scaled. In each violin plot, the mediation and quartiles of SA PRS within the group is displayed in red dot and the extended bar. Statistical significance of the SA vs. no SA/SI group differences (after multiple testing correction) was tested using *ggplot geom_signif*.

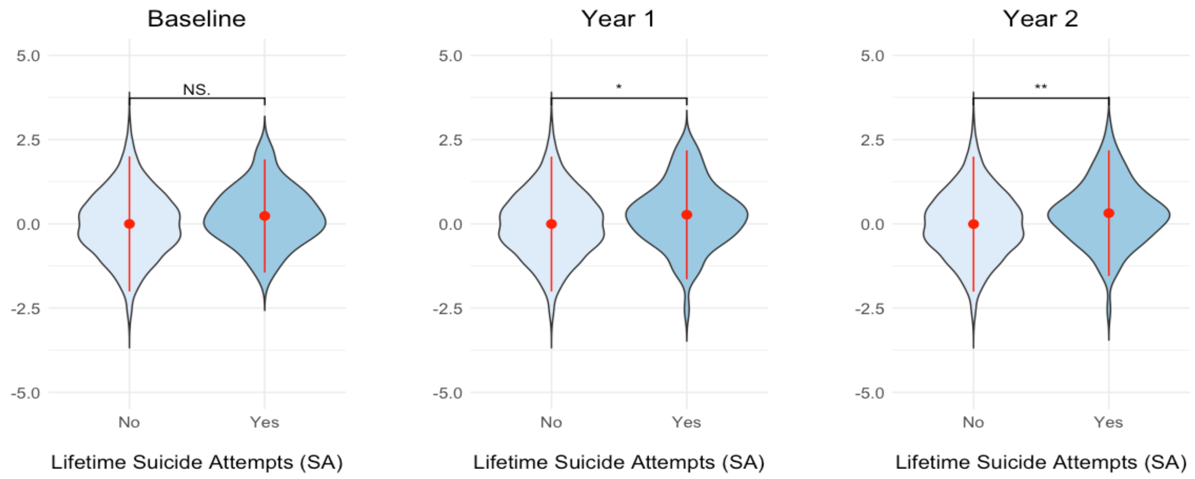

© 2022 Lee et al. *JAMA Psychiatry*.

eFigure 4. Beeswarm plot of SA PRSs in Year 2. Y-axis represents SA PRSs, which were regressed out for top ten genetic components and scaled. Individual dots represent each ABCD participant (blue: no SI/SA N=4,242, red: SA, N=102).

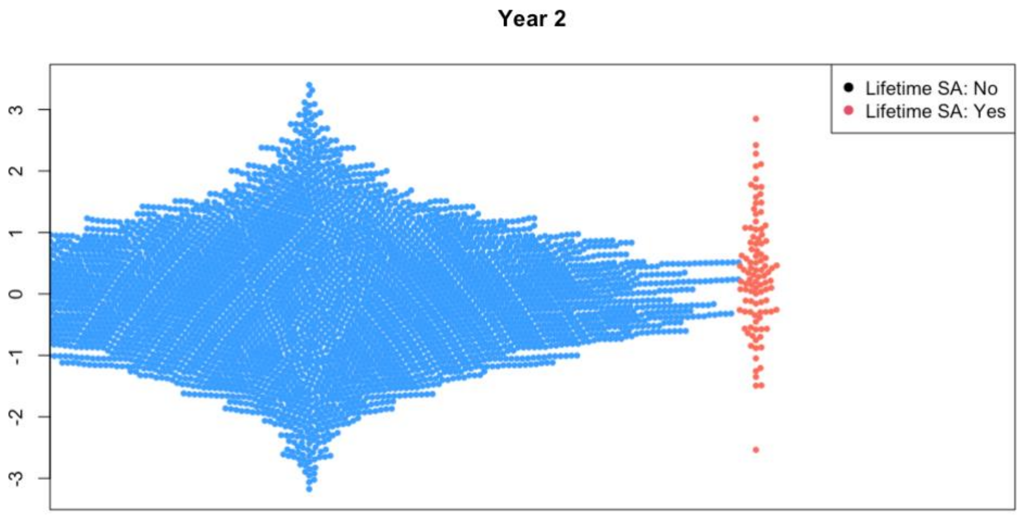

© 2022 Lee et al. *JAMA Psychiatry*.

eFigure 5. Beeswarm plot of SA PRSs in Year 2, with the same number of SA vs. no SA/SI participants. To intuitively compare the two groups with unbalanced size, we randomly selected 102 ABCD participants with no SI/SA experience, who have the matching propensity scores with 102 SA cases in year 2. Pre-calculated propensity scores were downloaded from the ABCD ACS (American Community Survey) Post Stratification Weights data (https://github.com/ABCD-STUDY/abcd_acs_raked_propensity). ABCD children with similar propensity scores share similar demographic and socio-economic covariates. Using the data, we generated two beeswarm plots shown below. On the y-axis, the first panel shows the SA PRS scores for the 204 participants, while the second panel displays the propensity scores for the same individuals.

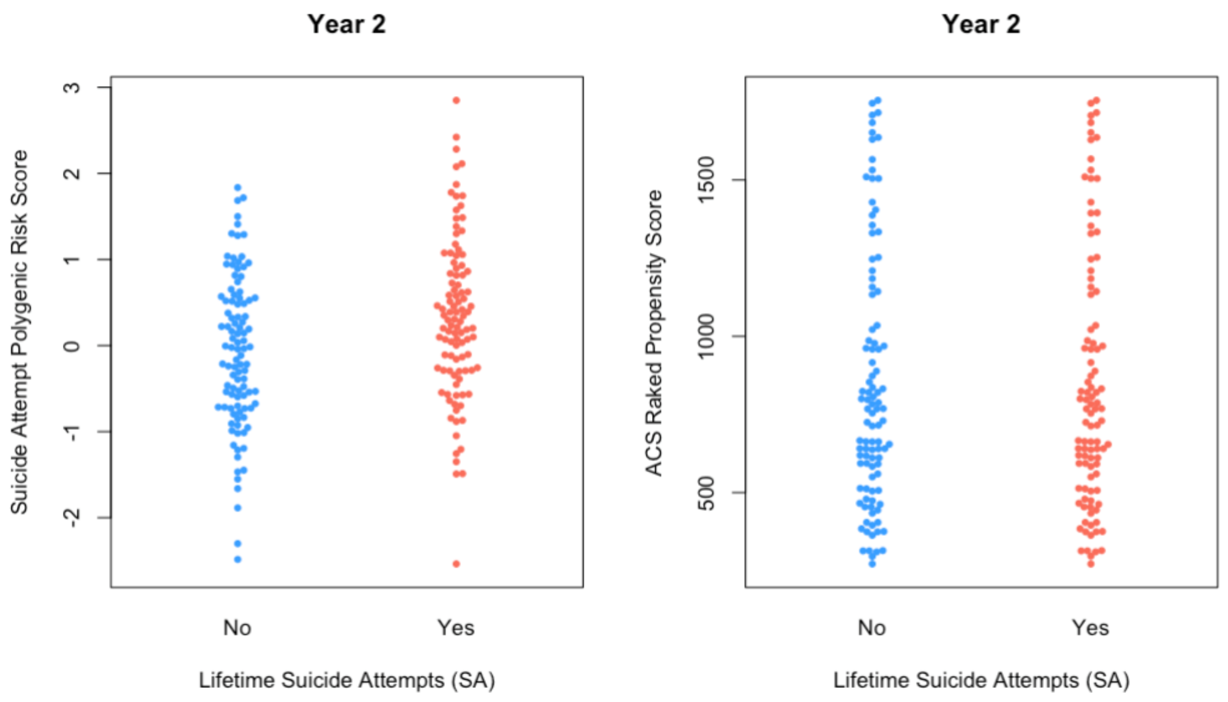

© 2022 Lee et al. *JAMA Psychiatry*.

eFigure 6. Sensitivity analysis results of children's suicide attempt (SA) polygenic risk scores (PRSs) and their lifetime suicidal ideation. Y-axis represents *Nagelkerke*'s pseudo-R^2, percentage of total variance explained by SA PRSs, controlling for age, sex, and top ten principal components covariates. X-axis represents ten p-value thresholds used to generate SA PRSs. A shade of green color represents the p-value of estimated beta coefficients of each PRS estimated in the logistic model. Significance was marked on the top of each bar following regression: $p \leq 1x10^{-3}$: '***', $0.001 < p \leq 0.01$: '**', $0.01 < p \leq 0.05$: '*'.

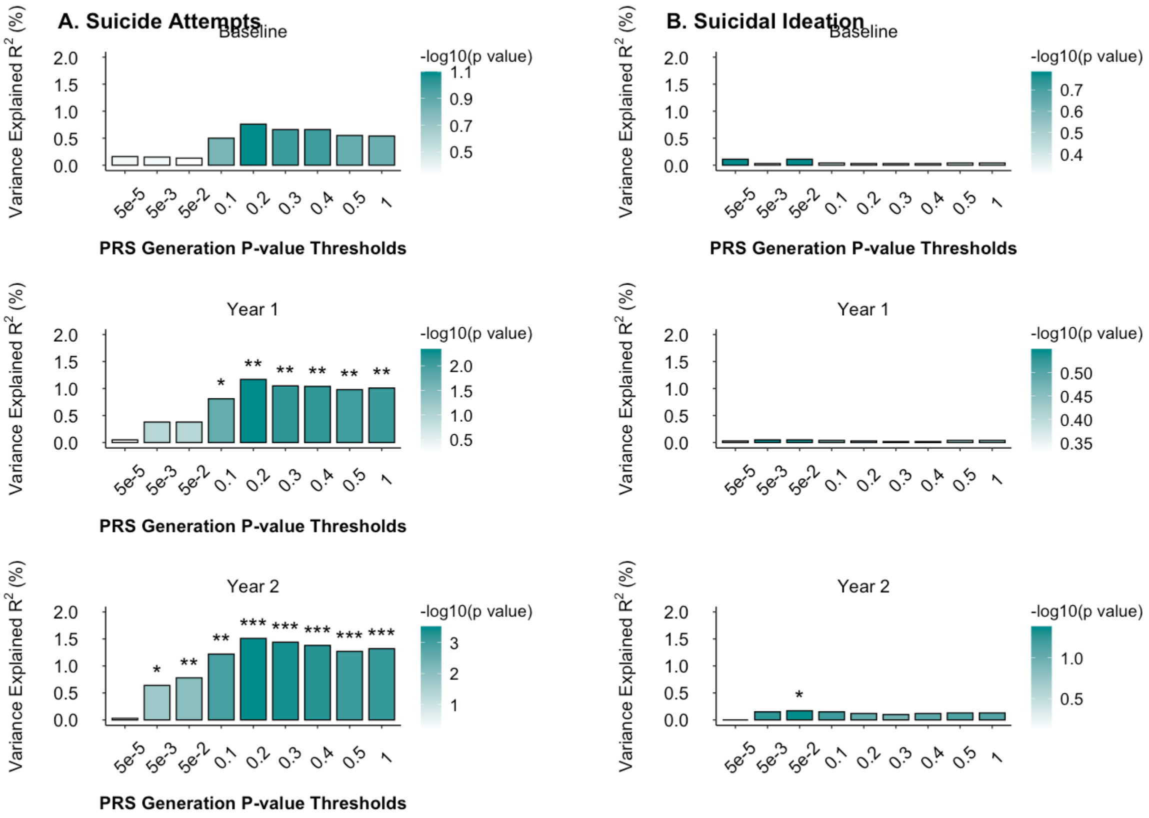

© 2022 Lee et al. *JAMA Psychiatry.*

eFigure 7. Correlations between 30 predictors used in lasso regression. The 34 risk predictors belonged to 6 categories: (1) demographics (age, sex); (2) socioeconomic status (single parent status, parents' lack of college education, poverty, and household income); (3) family history (parents' mental problems, depression, and suicide); (4) child psychopathology (8 CBCL syndromic normalized t-scores); (5) child temperament (10 EATQ measures) and (6) child genetic risk (MDD, ADHD, and SA PRSs). Notations from the top: single.parents (binary, single parent status), poverty (binary, household annual income < $20K), parental_scd_p_any (binary, whether any parent has a history of suicide), parental_ma_p_any (binary, whether any parent has a history of mental/emotional problems), parental_dprs_p_any (binary, whether any parent has a history of depression), sa_prs (quantitative, suicide attempt PRS), mdd_prs (quantitative, major depression PRS), adhd_prs (quantitative, ADHD PRS), eatqp2.fr (quantitative, frustration) eatqp2.ag (quantitative, aggression), cbcl_scr_syn_rulebreak_t (quantitative, rule-breaking behavior), cbcl_scr_syn_ aggressive _t (quantitative, aggressive behaviors), cbcl_scr_syn_thought_t (quantitative, thought problems), cbcl_scr_syn_social_t (quantitative, social problems), cbcl_scr_syn_attention_t (quantitative, attention problems), eatqp2.fe (quantitative, fear), cbcl_scr_syn_somatic_t (quantitative, somatic complaints), eatqp2.dp (quantitative, child temperament - depressive mood), cbcl_scr_syn_anxdep_t (quantitative, anxious depresion), cbcl_scr_syn_withdep_t (quantitative, withdrawal depression), SEX (binary, females=1), eatqp2.ac (quantitative, child temperament – activation control), eatqp2.hi (quantitative, child temperament – high-intensity pleasure/surgency), eatqp2.af (quantitative, child temperament - affiliation), eatqp2.sh (quantitative, child temperament - shy), eatqp2.at (quantitative, child temperament - attention), eatqp2.ic (quantitative, child temperament – inhibitory control), AGE (quantitative, year 2 age in year), parental.education.college (binary, whether parents engaged/completed college education), and household.income (ordinal, 4 categories bases on incomes).

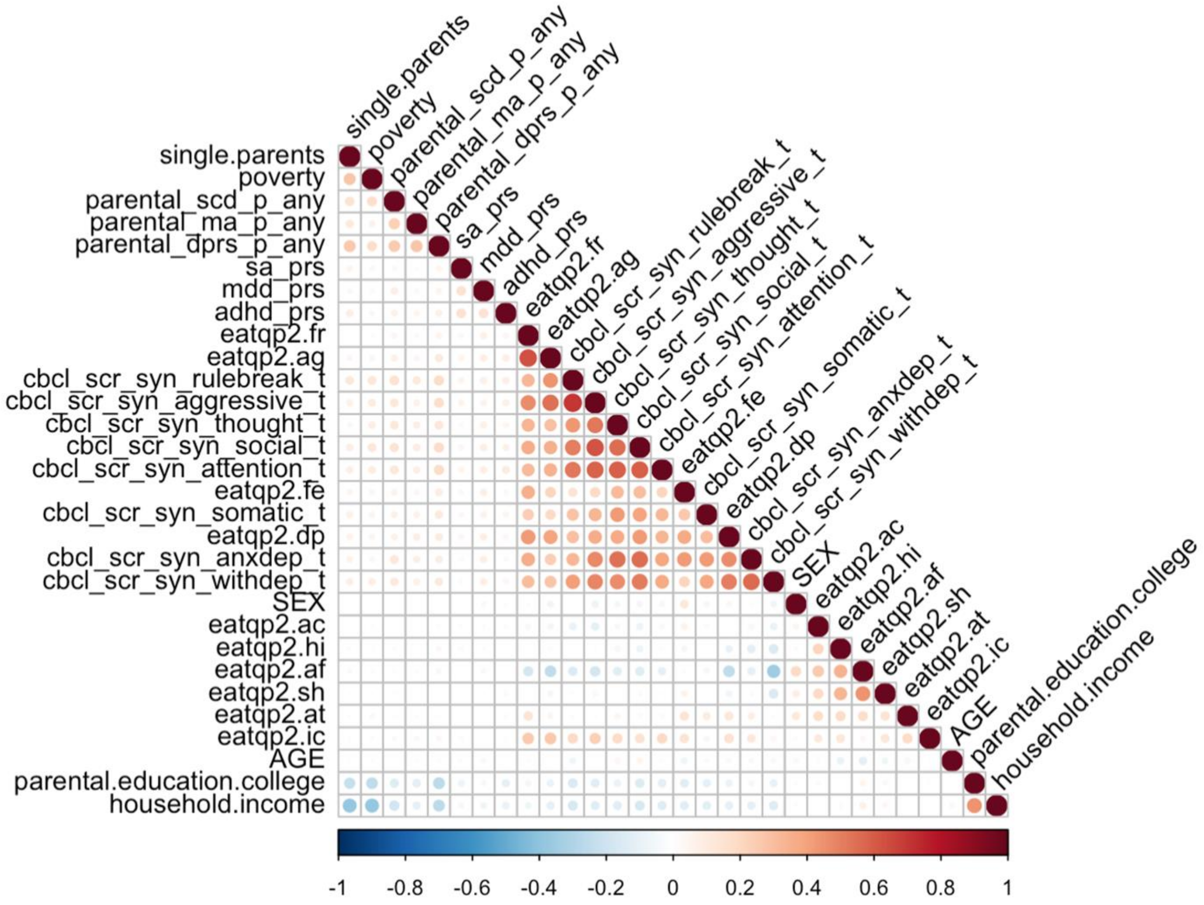

© 2022 Lee et al. *JAMA Psychiatry.*

eTable 1. Details of K-SADS-5 variables used for constructing lifetime suicide risk outcome measures. SA: suicide attempts, SI: suicidal ideation

Outcome	KSADS-5 Items	ABCD KSADS-5 Descriptions
SA	ksads_23_952_t	Diagnosis - Interrupted Attempt, Present
	ksads_23_953_t	Diagnosis - Aborted Attempt, Present
	ksads_23_954_t	Diagnosis - Suicide Attempt, Present
	ksads_23_963_t	Diagnosis - Interrupted Attempt, Past
	ksads_23_964_t	Diagnosis - Aborted Attempt, Past
	ksads_23_965_t	Diagnosis - Suicide Attempt, Past
SI	ksads_23_946_t	Diagnosis - Suicidal ideation Passive, Present
	ksads_23_947_t	Diagnosis - Suicidal ideation Active on specific, Present
	ksads_23_948_t	Diagnosis - Suicidal ideation Active method, Present
	ksads_23_949_t	Diagnosis - Suicidal ideation Active intent, Present
	ksads_23_950_t	Diagnosis - Suicidal ideation Active plan, Present
	ksads_23_951_t	Diagnosis - Preparatory Actions toward imminent Suicidal behavior, Present
	ksads_23_957_t	Diagnosis - Suicidal ideation Passive, Past
	ksads_23_958_t	Diagnosis - Suicidal ideation Active on specific, Past
	ksads_23_959_t	Diagnosis - Suicidal ideation Active method, Past
	ksads_23_960_t	Diagnosis - Suicidal ideation Active intent, Past
	ksads_23_961_t	Diagnosis - Suicidal ideation Active plan, Past
	ksads_23_962_t	Diagnosis - Preparatory Actions toward imminent Suicidal behavior, Past

© 2022 Lee et al. *JAMA Psychiatry*.

eTable 2. Correlations between SA PRSs and key demographic, andromorphic, ABCD study sites, and top ten genetic principal components (PC)

Category	Variables	Covariates	Correlation	Test Statistic	Sample No	P-value
DEMO	AGE	PC, Site	-0.01	-0.45	4344	6.53E-01
DEMO	SEX	PC, Site	0.01	0.56	4344	5.73E-01
ANTHRO	weight	Age, Sex, PC, Site	0.00	0.10	3747	9.17E-01
ANTHRO	height	Age, Sex, PC, Site	0.00	-0.13	4343	8.97E-01
ANTHRO	BMI	Age, Sex, PC, Site	0.02	0.91	3742	3.61E-01
Study Site	SITE_4	PC	0.01	0.57	4344	5.71E-01
Study Site	SITE_13	PC	0.02	1.09	4344	2.78E-01
Study Site	SITE_2	PC	0.00	-0.05	4344	9.60E-01
Study Site	SITE_17	PC	-0.01	-0.66	4344	5.07E-01
Study Site	SITE_20	PC	0.00	0.16	4344	8.72E-01
Study Site	SITE_9	PC	0.00	0.04	4344	9.72E-01
Study Site	SITE_8	PC	-0.02	-1.16	4344	2.46E-01
Study Site	SITE_21	PC	-0.01	-0.69	4344	4.91E-01
Study Site	SITE_6	PC	-0.01	-0.78	4344	4.35E-01
Study Site	SITE_15	PC	0.02	1.29	4344	1.97E-01
Study Site	SITE_19	PC	0.02	1.51	4344	1.31E-01
Study Site	SITE_10	PC	0.00	-0.30	4344	7.65E-01
Study Site	SITE_1	PC	0.01	0.75	4344	4.50E-01
Study Site	SITE_14	PC	-0.02	-1.08	4344	2.82E-01
Study Site	SITE_3	PC	0.00	-0.25	4344	8.04E-01
Study Site	SITE_11	PC	0.02	1.36	4344	1.73E-01
Study Site	SITE_18	PC	-0.01	-0.51	4344	6.07E-01
Study Site	SITE_22	PC	0.00	0.32	4344	7.51E-01
Study Site	SITE_5	PC	0.01	0.80	4344	4.24E-01
Study Site	SITE_12	PC	0.01	0.73	4344	4.65E-01
Study Site	SITE_7	PC	0.02	1.59	4344	1.12E-01
PC	PC1	NA	0.01	0.98	4344	3.25E-01
PC	PC2	NA	0.03	1.89	4344	5.92E-02
PC	PC3	NA	0.03	1.81	4344	7.08E-02
PC	PC4	NA	0.01	0.86	4344	3.88E-01
PC	PC5	NA	0.00	-0.21	4344	8.33E-01
PC	PC6	NA	0.01	0.51	4344	6.09E-01
PC	PC7	NA	0.03	1.71	4344	8.76E-02
PC	PC8	NA	-0.01	-0.38	4344	7.06E-01
PC	PC9	NA	0.02	1.54	4344	1.22E-01
PC	PC10	NA	0.00	-0.32	4344	7.52E-01

© 2022 Lee et al. *JAMA Psychiatry*.

eTable 3. Summary of ABCD variables used as predictors in multiple logistic regression for suicide risk outcome

Category	Variable	ABCD field	ABCD Survey	File Name
Demographics	Age	interview_age	Parent Demographics Survey	pdem02
	Sex	sex		
Socioeconomic Status	Parental Education Level	demo_prnt_ed_v2		
		demo_prtnr_ed_v2		
	Household Income Level	demo_comb_income_v2		
	Poverty Status			
	Parent Marital Status	demo_prnt_marital_v2		
Child Psychopathology	Anxious/Depressed	cbcl_scr_syn_anxdep_t	Parent Child Behavior Checklist Scores Aseba (CBCL)	abcd_cbcls01
	Withdrawn/Depressed	cbcl_scr_syn_withdep_t		
	Somatic Complaints	cbcl_scr_syn_somatic_t		
	Social Problems	cbcl_scr_syn_social_t		
	Thought Problems	cbcl_scr_syn_thought_t		
	Attention Problems	cbcl_scr_syn_attention_t		
	Rule-Breaking Behavior	cbcl_scr_syn_rulebreak_t		
	Aggressive Behavior	cbcl_scr_syn_aggressive_t		
	Internalizing Problems	cbcl_scr_syn_internal_t		
	Externalizing Problems	cbcl_scr_syn_external_t		
	Total Problems	cbcl_scr_syn_totprob_t		
Family History	Parental History of Suicide	famhx_ss_fath_prob_scd_p	Parent Family History Summary Scores	abcd_fhxssp01
		famhx_ss_moth_prob_scd_p		
	Parental History of Depression	famhx_ss_fath_prob_dprs_p		
		famhx_ss_moth_prob_dprs_p		
	Parental History of Emotional/Mental Problems	famhx_ss_fath_prob_prf_p		
		famhx_ss_moth_prob_prf_p		
Cognition	Vocabulary	nihtbx_picvocab_uncorrected	Youth NIH TB Summary Scores	abcd_tbss01
	Attention	nihtbx_flanker_uncorrected		
	Working memory	nihtbx_list_uncorrected		
	Executive function	nihtbx_cardsort_uncorrected		
	Overall cognition	nihtbx_totalcomp_uncorrected		

© 2022 Lee et al. *JAMA Psychiatry*.

eTable 4. Summary of ABCD variables tested for potential mediators in causal mediation analysis

Category	Variable	ABCD field	ABCD file
Psychopathology	Aggressive behavior	cbcl_scr_syn_aggressive_r	abcd_cbcls01
	Anxious depressed	cbcl_scr_syn_anxdep_r	
	Attention problems	cbcl_scr_syn_attention_r	
	Rule-breaking behavior	cbcl_scr_syn_rulebreak_r	
	Social problems	cbcl_scr_syn_social_r	
	Somatic complaints	cbcl_scr_syn_somatic_r	
	Thought problems	cbcl_scr_syn_thought_r	
	Withdrawn depressed	cbcl_scr_syn_withdep_r	
Personality / Temperament	Activation Control	eatq_finish_p, eatq_deal_p, eatq_before_hw_p, eatq_right_away_p,eatq_finish_hw_p, eatq_early_start_p eatq_puts_off_p	abcd_eatqp01.txt
	Affiliation	eatq_care_p, eatq_share_p, eatq_spend_time_p eatq_hugs_p, eatq_close_rel_p,eatq_friendly_p,	
	Aggression	eatq_insult_p, eatq_angry_hit_p, eatq_rude_p, eatq_blame_p, eatq_doorslam_p, eatq_makes_fun_p, eatq_no_criticize_p	
	Attention	eatq_concentrate_p, eatq_distracted_p, eatq_try_focus_p, eatq_peripheral_p, eatq_sidetracked_p, eatq_close_attention_p	
	Depressive Mood	eatq_enjoy_p, eatq_cry_p, eatq_sad_p, eatq_hardly_sad_p eatq_seems_sad_p	
	Fear	eatq_trouble_p, eatq_worry_p, eatq_attachment_p,eatq_ball_scared_p eatq_dark_scared_p eatq_alone_p,	
	Frustration	eatq_annoyed_p, eatq_irritated_crit_p,eatq_irritated_place_p eatq_irritated_enjoy_p eatq_disagree_p, eatq_frustrated_p	
	High-Intensity Pleasure/Surgency	eatq_africa_p, eatq_ski_slope_p, eatq_city_move_p, eatq_sea_dive_p, eatq_travel_p, eatq_race_car_p, eatq_s	
	Inhibitory Control	eatq_turn_taking_p, eatq_open_present_p,eatq_impulse_p, eatq_laugh_control_p eatq_stick_to_plan_p	
	Shyness	eatq_social_p, eatq_is_shy_p, eatq_not_shy_p,eatq_meet_p, eatq_shy_meet_p	

© 2022 Lee et al. *JAMA Psychiatry*.

eTable 5. List of data and methods used in the present study

Resource	Data Sources	Reference
ABCD v4.0	https:// data-archive.nimh.nih.gov/abcd	[20-22]
SA GWAS	https://tinyurl.com/ISGC2021	Mullin et al. (2022)[18]
Depression GWAS	https://www.med.unc.edu/pgc/download-results/	Howard et al. (2019)[7]
ADHD GWAS	https://www.med.unc.edu/pgc/download-results/	Demontis et al. (2019)[8]
CBCL	https://nda.nih.gov/data_structure.html?short_name=abcd_cbcls01	Achenbach T (2019)[9]
EATQ	https://nda.nih.gov/data_structure.html?short_name=abcd_eatqp01	Ellis and Rothbart (2001)[10]
ABCD K-SADS-PL DSM-5	https://nda.nih.gov/data_structure.html?short_name=abcd_yksad01	Kaufman et al. (2000)[4]
1000 Genomes Project	https://www.internationalgenome.org/data-portal/data-collection	Auton et al. (2015)[11]
Michigan Imputation Server	https://imputationserver.sph.umich.edu/	Das et al. (2016)[12]
minimac	https://genome.sph.umich.edu/wiki/Minimac4	Howie et al. (2012)[13]
Haplotype Reference Consortium	http://www.haplotype-reference-consortium.org/	McCarthy et al. (2016)[14]
PLINK	https://www.cog-genomics.org/plink/	Chang et al. (2015)[15]
PRSice	https://www.prsice.info/	Choi et al. (2019)[16]
glmnet	https://glmnet.stanford.edu/articles/glmnet.html	Friedman et al. (2010)[17]
mediation	http://CRAN.R-project.org/package=mediation	Tingley et al. (2019)[18]
glm	https://cran.r-project.org/doc/manuals/r-release/fullrefman.pdf	R Core Team (2020)[19]
Nagelkerke's pseudo-R^2	https://search.r-project.org/CRAN/refmans/DescTools/html/PseudoR2.html	Nagelkerke (1991)[20]

© 2022 Lee et al. *JAMA Psychiatry*.

eTable 6. Prevalence of children with suicide attempts (SAs), suicidal ideation (SI), and no SA/SI experiences over three consecutive years. Note that when participants reported both SAs and SI, we classify them into the SA group so that the two case groups do not overlap.

Outcome	SA	SI	CTRL	Enrollment Age (SD)
Baseline	37 (0.85%)	333 (7.67%)	3974 (91.48%)	9.93 (0.62)
Year 1	74 (1.70%)	488 (11.23%)	3782 (87.06%)	
Year 2	102 (2.35%)	601 (13.84%)	3641 (83.82%)	

© 2022 Lee et al. *JAMA Psychiatry*.

eTable 7. Distinct characteristics of children with suicide attempts (SAs), suicidal ideation (SI), and no SA/SI experiences in Year 1. We tested 25 variables in the domains of socioeconomic backgrounds, parental history of mental and suicide, neurocognition, temperament, and child psychopathology. P-values were marked in bold if they are significant after multiple testing correction (q<0.05).

Category	Variable	SA	SI	CTRL	SA.vs.CTRL	SI.vs.CTRL
		**74 (1.70%)**	**488 (11.23%)**	**3782 (87.06%)**		
Demo	Age	10.01 (0.65)	9.91 (0.62)	9.94 (0.62)	3.36E-01	3.24E-01
	Sex	32 (43.24%)	223 (45.70%)	1790 (47.33%)	5.62E-01	5.28E-01
SES	Poverty	9 (12.86%)	22 (4.76%)	137 (3.78%)	**3.80E-04**	3.66E-01
	Parents Not Married	29 (39.73%)	107 (21.97%)	637 (16.85%)	**6.93E-07**	**6.16E-03**
	Parents College Degree	37 (50.00%)	351 (71.93%)	2926 (77.39%)	**6.91E-08**	**8.51E-03**
Parental History	Depression	19 (25.68%)	66 (13.52%)	336 (8.88%)	**2.09E-06**	**1.28E-03**
	Mental/Emotional Problems	10 (13.51%)	32 (6.56%)	168 (4.44%)	**6.66E-04**	4.91E-02
	Suicide	19 (25.68%)	52 (10.66%)	189 (5.00%)	**4.75E-14**	**5.93E-07**
CBCL (Syndrome)	Anxious/Depressed	14 (19.44%)	33 (6.90%)	89 (2.43%)	**5.66E-17**	**1.15E-07**
	Withdrawl/Depresed	13 (18.06%)	30 (6.28%)	78 (2.13%)	**1.05E-16**	**1.97E-07**
	Somatic Problems	8 (11.11%)	20 (4.18%)	90 (2.45%)	**2.91E-05**	3.93E-02
	Social Problems	6 (8.33%)	13 (2.72%)	35 (0.95%)	**7.38E-08**	**1.55E-03**
	Thought Problems	15 (20.83%)	41 (8.58%)	151 (4.12%)	**6.61E-11**	**2.18E-05**
	Attention Problems	11 (15.28%)	22 (4.60%)	97 (2.65%)	**2.21E-09**	**2.36E-02**
	Rule-breaking Behavior	11 (15.28%)	14 (2.93%)	41 (1.12%)	**4.87E-22**	**2.36E-03**
	Aggressive Behavior	8 (11.11%)	18 (3.77%)	53 (1.45%)	**2.84E-09**	**4.85E-04**
	Internal Problems	14 (19.44%)	35 (7.32%)	108 (2.95%)	**8.17E-14**	**1.61E-06**
	External Problems	11 (15.28%)	15 (3.14%)	52 (1.42%)	**9.07E-18**	**9.03E-03**
	Total Problems	17 (23.61%)	26 (5.44%)	84 (2.29%)	**1.23E-26**	**1.06E-04**
CBCL (DSM-5)	Affective Problems	17 (23.61%)	38 (7.95%)	104 (2.84%)	**1.61E-21**	**1.64E-08**
	Anxiety Problems	12 (16.67%)	34 (7.11%)	127 (3.46%)	**2.88E-08**	**1.72E-04**
	Somatic Problems	4 (5.56%)	22 (4.60%)	124 (3.38%)	4.98E-01	2.19E-01
	Attention Deficit/Hyperactivity Problems	8 (11.11%)	19 (3.97%)	100 (2.73%)	**1.18E-04**	1.65E-01
	Opposite Defiant Problems	9 (12.50%)	24 (5.02%)	91 (2.48%)	**1.25E-06**	**2.44E-03**
	Conduct Problems	10 (13.89%)	20 (4.18%)	49 (1.34%)	**1.40E-15**	**1.15E-05**

© 2022 Lee et al. *JAMA Psychiatry*.

eTable 8. Distinct characteristics of children with suicide attempts (SAs), suicidal ideation (SI), and no SA/SI experiences in Year 2. We tested 25 variables in the domains of socioeconomic backgrounds, parental history of mental and suicide, neurocognition, temperament, and child psychopathology. P-values were marked in bold if they are significant after multiple testing correction (q<0.05).

Category	Variable	SA	SI	CTRL	SA vs. Controls	SI vs. Controls
		102 (2.35%)	601 (13.84%)	3641 (83.82%)		
Demo	Age	10.02 (0.63)	9.91 (0.63)	9.93 (0.61)	1.73E-01	4.76E-01
	Sex	52 (50.98%)	288 (47.92%)	1705 (46.83%)	4.66E-01	6.51E-01
SES	Poverty	11 (11.46%)	25 (4.38%)	132 (3.78%)	**4.16E-04**	5.68E-01
	Parents Not Married	41 (40.59%)	130 (21.67%)	602 (16.54%)	**6.20E-10**	**2.54E-03**
	Parents No College Degree	54 (52.94%)	437 (72.71%)	2823 (77.55%)	**1.22E-08**	**1.06E-02**
Parental History	Parental History of Depression	21 (20.59%)	85 (14.14%)	315 (8.65%)	**6.78E-05**	**2.76E-05**
	Parental History of Mental Problems	13 (12.75%)	42 (6.99%)	155 (4.26%)	**1.22E-04**	**4.47E-03**
	Parental History of Suicide	20 (19.61%)	65 (10.82%)	175 (4.81%)	**1.47E-10**	**6.17E-09**
CBCL (syndrome)	Anxious/Depressed	13 (18.31%)	40 (8.64%)	56 (2.09%)	**1.58E-16**	**1.12E-13**
	Withdrawl/Depresed	7 (9.86%)	20 (4.32%)	40 (1.49%)	**9.08E-07**	**8.59E-05**
	Somatic Problems	10 (14.08%)	25 (5.40%)	61 (2.27%)	**5.87E-09**	**2.55E-04**
	Social Problems	7 (9.86%)	12 (2.59%)	22 (0.82%)	**1.22E-11**	**1.56E-03**
	Thought Problems	15 (21.13%)	30 (6.48%)	67 (2.50%)	**1.88E-18**	**9.25E-06**
	Attention Problems	6 (8.45%)	22 (4.75%)	51 (1.90%)	**6.62E-04**	**3.22E-04**
	Rule-breaking Behavior	6 (8.45%)	6 (1.30%)	11 (0.41%)	**7.73E-15**	3.95E-02
	Aggressive Behavior	11 (15.49%)	12 (2.59%)	15 (0.56%)	**2.31E-34**	**4.00E-05**
	Internal Problems	12 (16.90%)	33 (7.13%)	53 (1.97%)	**7.07E-15**	**9.01E-10**
	External Problems	9 (12.68%)	9 (1.94%)	13 (0.48%)	**8.48E-27**	**1.48E-03**
	Total Problems	12 (16.90%)	21 (4.54%)	31 (1.15%)	**6.75E-24**	**3.93E-07**
CBCL (DSM-5)	Affective Problems	17 (23.94%)	40 (8.64%)	52 (1.94%)	**9.56E-30**	**8.76E-15**
	Anxiety Problems	14 (19.72%)	39 (8.42%)	63 (2.35%)	**4.45E-17**	**2.46E-11**
	Somatic Problems	12 (16.90%)	27 (5.83%)	86 (3.20%)	**5.68E-09**	**7.57E-03**
	Attention Deficit/Hyperactivity Problems	6 (8.45%)	19 (4.10%)	55 (2.05%)	**1.33E-03**	**1.15E-02**
	Opposite Defiant Problems	9 (12.68%)	14 (3.02%)	38 (1.42%)	**1.31E-11**	**2.09E-02**
	Conduct Problems	9 (12.68%)	11 (2.38%)	21 (0.78%)	**3.49E-19**	**3.67E-03**

© 2022 Lee et al. *JAMA Psychiatry.*

eTable 9. Results of logistic regression analysis for suicide attempts (SA) polygenic risk scores (PRSs) on children's lifetime suicide risk outcome measures. To examine association of SA PRSs with lifetime suicide risk, we used multiple logistic regression *glm* in R. Lifetime suicide attempts (SAs) and suicidal ideation (SI) were used as binary dependent variables, while SA PRS was used as an independent variable along with age, sex, and top ten principal components of genetic ancestry as covariates. To measure the unique proportion of variance explained by PRSs, we calculated *Nagelkerke*'s pseudo-R^2.

PRS	Outcome	Event	Case No	Pseudo R2	Odds Ratio	95% Confidence Interval	Pvalue	Bonf.Pvalue	Bonf.Pvalue .Sig
SA	SA	baseline	37 (0.85%)	0.76	1.34	0.97-1.85	7.94E-02	4.76E-01	ns
		year1	74 (1.7%)	1.17	1.39	1.11-1.75	4.55E-03	2.73E-02	*
		year2	102 (2.35%)	1.51	1.43	1.18-1.75	3.09E-04	1.85E-03	**
	SI	baseline	333 (7.67%)	0.03	1.04	0.93-1.16	4.54E-01	1.00E+00	ns
		year1	488 (11.23%)	0.03	1.04	0.95-1.14	3.93E-01	1.00E+00	ns
		year2	601 (13.84%)	0.12	1.07	0.98-1.17	9.30E-02	5.58E-01	ns

© 2022 Lee et al. *JAMA Psychiatry*.

eTable 10. Sensitivity analysis results of suicide attempts (SA) polygenic risk scores (PRSs) on SA risk model with respect to different p-value thresholds. For SA PRSs of significant association with children's SA in year 2, we conducted sensitivity analyses to assess whether the identified association varies as the p-value threshold for constructing PRS changes. Nine p-values in total, $5x10^{-5}$, $5x10^{-3}$, $5x10^{-2}$, 0.1, 0.2, 0.3, 0.4, 0.5, and 1.0, were used in sensitivity analysis. Lifetime suicide attempts (SAs) were used as a binary dependent variable, while SA PRS was used as an independent variable along with age, sex, and top ten principal components of genetic ancestry as covariates. To measure the unique proportion of variance explained by PRSs, we calculated *Nagelkerke*'s pseudo-R^2.

PRS	Outcome	Event	Case No	PRS SCORE	Pseudo R2	Odds Ratio	nfidence Interv	Pvalue	Sig.Pvalue
SA	SA	baseline	37 (0.85%)	5.00E-05	0.16	1.14	0.82-1.59	4.27E-01	ns
				0.005	0.15	1.14	0.82-1.57	4.29E-01	ns
				0.05	0.13	1.13	0.82-1.56	4.70E-01	ns
				0.1	0.5	1.26	0.91-1.74	1.53E-01	ns
				0.2	0.76	1.34	0.97-1.85	7.94E-02	ns
				0.3	0.66	1.31	0.95-1.81	1.02E-01	ns
				0.4	0.66	1.31	0.95-1.81	1.03E-01	ns
				0.5	0.55	1.28	0.93-1.77	1.35E-01	ns
				1	0.54	1.27	0.92-1.76	1.40E-01	ns
		year1	74 (1.7%)	5.00E-05	0.05	1.07	0.85-1.35	5.69E-01	ns
				0.005	0.38	1.21	0.96-1.52	1.06E-01	ns
				0.05	0.38	1.21	0.96-1.52	1.07E-01	ns
				0.1	0.81	1.31	1.04-1.65	1.86E-02	*
				0.2	1.17	1.39	1.11-1.75	4.55E-03	**
				0.3	1.05	1.36	1.08-1.72	7.25E-03	**
				0.4	1.04	1.36	1.08-1.72	7.53E-03	**
				0.5	0.98	1.35	1.07-1.70	9.64E-03	**
				1	1.01	1.36	1.08-1.72	8.57E-03	**
		year2	102 (2.35%)	5.00E-05	0.03	1.05	0.86-1.28	6.30E-01	ns
				0.005	0.64	1.27	1.04-1.55	1.84E-02	*
				0.05	0.78	1.3	1.07-1.58	9.37E-03	**
				0.1	1.22	1.38	1.13-1.68	1.15E-03	**
				0.2	1.51	1.43	1.18-1.75	3.09E-04	***
				0.3	1.44	1.42	1.17-1.73	4.33E-04	***
				0.4	1.38	1.42	1.17-1.73	5.53E-04	***
				0.5	1.27	1.39	1.14-1.69	9.29E-04	***
				1	1.32	1.41	1.15-1.71	7.46E-04	***

© 2022 Lee et al. *JAMA Psychiatry.*

eTable 11. Sensitivity analysis results of suicide attempts (SA) polygenic risk scores (PRSs) on suicidal ideation (SI) risk model with respect to different p-value thresholds. Sensitivity analyses confirmed null association of SA PRSs with SI across different p-value thresholds for constructing PRS changes. Nine p-values in total, $5x10^{-5}$, $5x10^{-3}$, $5x10^{-2}$, 0.1, 0.2, 0.3, 0.4, 0.5, and 1.0, were used in analysis. Lifetime SI was used as a binary dependent variable, while SA PRS was used as an independent variable along with age, sex, and top ten principal components of genetic ancestry as covariates. To measure the unique proportion of variance explained by PRSs, we calculated *Nagelkerke*'s pseudo-R^2.

PRS	Outcome	Event	Case No	PRS SCORE	Pseudo R2	Odds Ratio	Confidence Interval	Pvalue	Sig.Pvalue
SA	SI	baseline	333 (7.67%)	5.00E-05	0.11	0.92	0.83-1.03	1.66E-01	ns
				0.005	0.03	1.04	0.93-1.17	4.92E-01	ns
				0.05	0.11	1.08	0.97-1.21	1.64E-01	ns
				0.1	0.04	1.05	0.94-1.18	3.86E-01	ns
				0.2	0.03	1.04	0.93-1.16	4.54E-01	ns
				0.3	0.03	1.04	0.93-1.17	4.70E-01	ns
				0.4	0.03	1.04	0.93-1.17	4.98E-01	ns
				0.5	0.04	1.05	0.94-1.18	3.96E-01	ns
				1	0.04	1.05	0.94-1.18	3.91E-01	ns
		year1	488 (11.23%)	5.00E-05	0.03	0.96	0.88-1.06	4.45E-01	ns
				0.005	0.05	1.05	0.96-1.16	2.82E-01	ns
				0.05	0.05	1.05	0.96-1.16	2.88E-01	ns
				0.1	0.04	1.05	0.96-1.16	3.49E-01	ns
				0.2	0.03	1.04	0.95-1.14	3.93E-01	ns
				0.3	0.02	1.04	0.95-1.14	4.67E-01	ns
				0.4	0.02	1.04	0.95-1.14	4.62E-01	ns
				0.5	0.04	1.04	0.95-1.14	3.81E-01	ns
				1	0.04	1.04	0.95-1.14	3.70E-01	ns
		year2	601 (13.84%)	5.00E-05	0	0.99	0.91-1.08	7.57E-01	ns
				0.005	0.15	1.09	1.00-1.19	5.39E-02	ns
				0.05	0.17	1.09	1.00-1.19	4.14E-02	*
				0.1	0.15	1.08	0.99-1.18	6.19E-02	ns
				0.2	0.12	1.07	0.98-1.17	9.30E-02	ns
				0.3	0.1	1.07	0.98-1.17	1.20E-01	ns
				0.4	0.12	1.08	0.99-1.18	8.65E-02	ns
				0.5	0.13	1.08	0.99-1.18	7.44E-02	ns
				1	0.13	1.08	0.99-1.18	7.80E-02	ns

© 2022 Lee et al. *JAMA Psychiatry*.

eTable 12. Results of logistic regression analysis for major depression (MD) and attention deficit/hyperactivity disorder (ADHD) polygenic risk scores (PRSs) on predicting children's lifetime suicide risk outcome measures. To examine association of ADHD and MD PRSs with lifetime suicide risk, we used multiple logistic regression *glm* in R. To measure the unique proportion of variance explained by PRSs, we calculated *Nagelkerke*'s pseudo-R^2.

Outcome	Event	Case No	PRS SCORE	MD			ADHD		
				OR	CI	Pvalue	OR	CI	Pvalue
SA	baseline	37 (0.85%)	5.00E-05	1.42	1.019-1.976	**3.81E-02**	1.57	1.13-2.17	**6.07E-03**
			0.005	1.65	1.185-2.303	**3.06E-03**	1.1	0.80-1.53	5.57E-01
			0.05	1.83	1.301-2.566	**5.10E-04**	1.1	0.79-1.54	5.38E-01
			0.1	1.68	1.199-2.356	**2.60E-03**	1.17	0.84-1.64	3.30E-01
			0.2	1.61	1.149-2.259	**5.67E-03**	1.15	0.83-1.60	3.91E-01
			0.3	1.48	1.053-2.067	**2.37E-02**	1.22	0.88-1.70	2.27E-01
			0.4	1.54	1.094-2.159	**1.32E-02**	1.2	0.86-1.67	2.84E-01
			0.5	1.47	1.049-2.068	**2.53E-02**	1.22	0.88-1.70	2.42E-01
			1	1.45	1.033-2.033	**3.19E-02**	1.23	0.89-1.72	2.11E-01
	year1	74 (1.7%)	5.00E-05	1.50	1.185-1.901	**7.53E-04**	1.25	0.99-1.57	6.81E-02
			0.005	1.49	1.179-1.885	**8.61E-04**	1.06	0.84-1.34	6.17E-01
			0.05	1.56	1.226-1.976	**2.75E-04**	1.31	1.04-1.66	**2.34E-02**
			0.1	1.53	1.202-1.938	**5.18E-04**	1.31	1.04-1.66	**2.15E-02**
			0.2	1.57	1.237-2.003	**2.21E-04**	1.25	0.98-1.58	6.83E-02
			0.3	1.50	1.177-1.906	**1.01E-03**	1.26	0.99-1.59	6.00E-02
			0.4	1.52	1.192-1.936	**7.34E-04**	1.25	0.98-1.58	6.46E-02
			0.5	1.48	1.163-1.889	**1.48E-03**	1.26	0.99-1.59	6.01E-02
			1	1.46	1.146-1.861	**2.18E-03**	1.26	0.99-1.59	5.92E-02
	year2	102 (2.35%)	5.00E-05	1.40	1.144-1.710	**1.06E-03**	1.19	0.97-1.44	9.68E-02
			0.005	1.53	1.250-1.871	**3.60E-05**	1.08	0.89-1.32	4.09E-01
			0.05	1.63	1.325-2.002	**3.59E-06**	1.32	1.08-1.62	**5.62E-03**
			0.1	1.60	1.299-1.964	**8.71E-06**	1.36	1.11-1.67	**2.42E-03**
			0.2	1.61	1.310-1.983	**6.52E-06**	1.27	1.04-1.55	**1.72E-02**
			0.3	1.53	1.240-1.877	**6.51E-05**	1.3	1.06-1.59	**1.14E-02**
			0.4	1.55	1.261-1.915	**3.60E-05**	1.3	1.06-1.59	**1.20E-02**
			0.5	1.53	1.240-1.884	**6.90E-05**	1.31	1.07-1.60	**9.28E-03**
			1	1.51	1.229-1.866	**9.73E-05**	1.3	1.06-1.59	**1.22E-02**
SI	baseline	333 (7.67%)	5.00E-05	1.00	0.895-1.122	9.71E-01	1.07	0.96-1.20	2.15E-01
			0.005	1.03	0.923-1.156	5.76E-01	1.09	0.98-1.22	1.06E-01
			0.05	1.04	0.925-1.161	5.36E-01	1.16	1.04-1.30	**1.22E-02**
			0.1	1.06	0.945-1.186	3.27E-01	1.15	1.03-1.29	**1.28E-02**
			0.2	1.05	0.935-1.174	4.24E-01	1.13	1.01-1.26	**3.59E-02**
			0.3	1.05	0.937-1.177	3.97E-01	1.12	1.00-1.25	**5.09E-02**
			0.4	1.03	0.920-1.155	6.04E-01	1.14	1.02-1.28	**2.44E-02**
			0.5	1.02	0.913-1.147	6.97E-01	1.14	1.02-1.28	**2.08E-02**
			1	1.03	0.915-1.149	6.66E-01	1.14	1.02-1.28	**2.25E-02**
	year1	488 (11.23%)	5.00E-05	1.04	0.942-1.139	4.68E-01	1.05	0.96-1.16	3.47E-01
			0.005	1.08	0.978-1.183	1.34E-01	1.08	0.98-1.19	1.07E-01
			0.05	1.05	0.956-1.157	3.01E-01	1.16	1.06-1.28	**2.37E-03**
			0.1	1.07	0.975-1.181	1.48E-01	1.19	1.08-1.30	**6.66E-04**
			0.2	1.05	0.953-1.154	3.33E-01	1.16	1.05-1.28	**1.95E-03**
			0.3	1.06	0.963-1.167	2.36E-01	1.15	1.04-1.27	**5.25E-03**
			0.4	1.05	0.952-1.153	3.42E-01	1.16	1.05-1.28	**2.25E-03**
			0.5	1.05	0.951-1.152	3.54E-01	1.16	1.05-1.28	**1.90E-03**
			1	1.05	0.956-1.159	2.98E-01	1.15	1.04-1.27	**3.23E-03**
	year2	601 (13.84%)	5.00E-05	1.02	0.937-1.114	6.23E-01	1.07	0.98-1.17	1.02E-01
			0.005	1.06	0.973-1.158	1.78E-01	1.07	0.98-1.17	**9.95E-02**
			0.05	1.06	0.966-1.151	2.34E-01	1.16	1.06-1.27	**6.07E-04**
			0.1	1.07	0.980-1.168	1.29E-01	1.16	1.06-1.27	**9.29E-04**
			0.2	1.06	0.971-1.157	1.95E-01	1.15	1.05-1.26	**2.29E-03**
			0.3	1.06	0.973-1.160	1.74E-01	1.14	1.04-1.24	**5.11E-03**
			0.4	1.05	0.961-1.146	2.86E-01	1.15	1.05-1.26	**1.43E-03**
			0.5	1.05	0.960-1.144	2.95E-01	1.15	1.05-1.26	**1.60E-03**
			1	1.05	0.961-1.146	2.82E-01	1.14	1.04-1.24	**2.71E-03**

© 2022 Lee et al. *JAMA Psychiatry*.

eTable 13. Logistic regression analysis results of suicide attempts (SA) polygenic risk scores (PRSs) on children's lifetime SA outcome measures, conditioning on major depression (MD) and attention deficit/hyperactivity disorder (ADHD) PRSs. We conducted two-PRS logistic regression analyses to assess whether the identified association between SA PRSs and children's SA stays significant when PRSs for MD and ADHD, two psychiatric disorders of strong association with children's suicidal behaviors, were added in the same model. Lifetime suicide attempts (SAs) were used as a binary dependent variable, while SA, MD, and ADHD PRSs were used as independent variables along with age, sex, and top ten principal components of genetic ancestry as covariates. To measure the unique proportion of variance explained by PRSs, we calculated *Nagelkerke*'s pseudo-R^2.

Outcome	Event	PRS1	PRS2	Case No	PRS SCORE	PRS1					PRS2		
						Pseudo R2	OR	95% CI	Pvalue	Sig Pvalue	OR	95% CI	Pvalue
SA	Year 1	SA	ADHD	74 (1.7%)	0.1	0.54	1.26	1.00-1.59	5.48E-02	ns	1.35	1.071-1.707	1.12E-02
					0.2	0.92	1.35	1.07-1.70	1.22E-02	*	1.29	1.018-1.625	3.45E-02
					0.3	0.81	1.32	1.05-1.67	1.84E-02	*	1.29	1.022-1.633	3.23E-02
					0.4	0.8	1.32	1.05-1.67	1.95E-02	*	1.29	1.023-1.635	3.15E-02
					0.5	0.74	1.31	1.04-1.65	2.44E-02	*	1.29	1.023-1.635	3.14E-02
					1	0.77	1.31	1.04-1.65	2.13E-02	*	1.29	1.021-1.631	3.32E-02
			MD	74 (1.7%)	0.1	0.43	1.22	0.97-1.54	8.72E-02	ns	1.54	1.210-1.953	4.25E-04
					0.2	0.66	1.28	1.02-1.62	3.37E-02	*	1.56	1.225-1.984	3.07E-04
					0.3	0.62	1.28	1.02-1.62	3.87E-02	*	1.49	1.172-1.898	1.14E-03
					0.4	0.61	1.28	1.02-1.62	4.00E-02	*	1.52	1.191-1.933	7.44E-04
					0.5	0.59	1.27	1.01-1.61	4.42E-02	*	1.48	1.165-1.890	1.41E-03
					1	0.63	1.28	1.02-1.62	3.69E-02	*	1.46	1.147-1.860	2.14E-03
SA	Year 2	SA	ADHD	102 (2.35%)	0.1	0.83	1.31	1.07-1.60	7.20E-03	**	1.40	1.146-1.711	9.83E-04
					0.2	1.18	1.38	1.13-1.68	1.40E-03	**	1.32	1.080-1.613	6.62E-03
					0.3	1.11	1.36	1.12-1.66	1.92E-03	**	1.33	1.091-1.630	4.96E-03
					0.4	1.05	1.36	1.12-1.66	2.54E-03	**	1.34	1.096-1.638	4.25E-03
					0.5	0.95	1.34	1.09-1.63	4.19E-03	**	1.35	1.105-1.651	3.36E-03
					1	1.01	1.35	1.11-1.65	3.14E-03	**	1.33	1.091-1.630	4.99E-03
			MD	102 (2.35%)	0.1	0.68	1.28	1.05-1.57	1.50E-02	*	1.61	1.306-1.974	7.19E-06
					0.2	0.87	1.32	1.08-1.62	5.89E-03	**	1.60	1.296-1.963	1.04E-05
					0.3	0.89	1.32	1.08-1.62	5.40E-03	**	1.52	1.234-1.868	7.84E-05
					0.4	0.84	1.32	1.08-1.62	6.83E-03	**	1.55	1.261-1.914	3.46E-05
					0.5	0.77	1.3	1.06-1.58	9.61E-03	**	1.54	1.246-1.891	5.71E-05
					1	0.84	1.32	1.08-1.62	7.13E-03	**	1.52	1.231-1.868	9.00E-05

eTable 14. Mediation analysis results between SA PRSs, mediator variables, and SA. For each mediator variable, univariate linear regression was conducted to examine its association with SA PRS. Multivariate logistic regression was then performed where SA PRSs and a mediator were used as independent variables for predicting the SA outcome. In all regression, age, sex, and top ten genetic principal components were covaried. ACME: average causal mediation effect, ADE: average direct effect. MP: mediated proportion. Long table is displayed separately on the top and bottom. Variable names stand for: eatqp2.fr (quantitative, frustration) eatqp2.ag (quantitative, aggression), cbcl_scr_syn_rulebreak_t (quantitative, rule-breaking behavior), cbcl_scr_syn_ aggressive _t (quantitative, aggressive behaviors), cbcl_scr_syn_thought_t (quantitative, thought problems), cbcl_scr_syn_social_t (quantitative, social problems), cbcl_scr_syn_attention_t (quantitative, attention problems), eatqp2.fe (quantitative, fear), cbcl_scr_syn_somatic_t (quantitative, somatic complaints), eatqp2.dp (quantitative, child temperament - depressive mood), cbcl_scr_syn_anxdep_t (quantitative, anxious depresion), cbcl_scr_syn_withdep_t (quantitative, withdrawal depression), eatqp2.ac (quantitative, child temperament – activation control), eatqp2.hi (quantitative, child temperament – high-intensity pleasure/surgency), eatqp2.af (quantitative, child temperament - affiliation), eatqp2.sh (quantitative, child temperament - shy), eatqp2.at (quantitative, child temperament - attention), eatqp2.ic (quantitative, child temperament – inhibitory control).

Mediator		SA PRS to Mediator (univariate linear regression)				SA PRS & Mediator on SA (multivariate logistic regression)						Mediation Effects			
						Mediator			SA PRS						
Instrument	ABCD Variable	OR	CI	p	sig	OR	CI (95%)	P	OR	CI (95%)	P	MP	CI (95%)	ACME.p	ADE.p
CBCL	cbcl_scr_syn_aggressive_t	1.06	1.03-1.10	4.36E-04	***	1.72	1.51-1.96	3.54E-16	1.32	1.04-1.68	2.44E-02	0.11	0.04-0.43	**<2e-16**	0.004
	cbcl_scr_syn_anxdep_t	1.03	1.00-1.07	6.67E-02	ns	1.94	1.68-2.23	<2e-16	1.39	1.09-1.77	7.22E-03	0.06	-0.22	0.056	0.004
	cbcl_scr_syn_attention_t	1.06	1.02-1.10	9.58E-04	***	1.66	1.44-1.91	2.33E-12	1.33	1.05-1.70	2.01E-02	0.09	0.03-0.40	0.004	0.02
	cbcl_scr_syn_rulebreak_t	1.05	1.02-1.09	2.91E-03	**	1.76	1.54-2.01	<2e-16	1.35	1.05-1.72	1.71E-02	0.09	0.03-0.43	0.004	0.016
	cbcl_scr_syn_social_t	1.05	1.01-1.09	5.27E-03	**	1.82	1.59-2.08	<2e-16	1.34	1.04-1.71	2.09E-02	0.09	0.02-0.39	0.008	0.004
	cbcl_scr_syn_somatic_t	1.04	1.01-1.08	1.57E-02	*	1.63	1.36-1.96	1.29E-07	1.37	1.08-1.74	1.05E-02	0.06	0.01-0.18	0.008	0.008
	cbcl_scr_syn_thought_t	1.03	1.00-1.07	7.61E-02	ns	2	1.70-2.35	<2e-16	1.37	1.08-1.75	1.08E-02	0.06	-0.25	0.064	<2e-16
	cbcl_scr_syn_withdep_t	1.03	0.99-1.06	1.46E-01	ns	1.72	1.49-1.99	1.74E-13	1.38	1.08-1.75	8.63E-03	0.04	-0.21	0.136	0.004
EATQ	eatqp2_ac	1.01	0.98-1.05	3.88E-01	ns	0.87	0.71-1.06	1.64E-01	1.38	1.12-1.69	2.43E-03	-0.01	-0.05	0.5	<2e-16
	eatqp2_af	0.99	0.96-1.02	5.44E-01	ns	0.74	0.61-0.90	2.91E-03	1.38	1.12-1.70	2.58E-03	0.01	-0.07	0.48	<2e-16
	eatqp2_ag	1.04	1.01-1.07	1.08E-02	*	1.88	1.55-2.28	1.43E-10	1.33	1.08-1.64	7.05E-03	0.08	0.02-0.26	0.008	0.004
	eatqp2_at	1	0.97-1.03	8.81E-01	ns	0.88	0.72-1.08	2.16E-01	1.37	1.12-1.69	2.61E-03	0	-0.06	0.928	0.008
	eatqp2_dp	1.04	1.01-1.07	1.05E-02	*	2.46	2.01-3.01	<2e-16	1.32	1.07-1.63	1.01E-02	0.12	0.03-0.47	**<2e-16**	0.016
	eatqp2_fe	1.03	1.00-1.06	7.27E-02	ns	1.71	1.40-2.10	2.16E-07	1.36	1.10-1.68	3.84E-03	0.05	-0.17	0.06	0.004
	eatqp2_fr	1.03	1.00-1.07	3.27E-02	*	1.84	1.49-2.29	2.81E-08	1.35	1.10-1.67	4.58E-03	0.06	0.00-0.17	0.048	0.004
	eatqp2_hi	1.01	0.98-1.04	6.91E-01	ns	0.69	0.56-0.84	2.35E-04	1.38	1.12-1.71	2.28E-03	-0.01	-0.09	0.708	0.004
	eatqp2_ic	1.02	0.99-1.05	1.78E-01	ns	1.39	1.13-1.71	1.56E-03	1.36	1.11-1.68	3.43E-03	0.02	-0.1	0.19	<2e-16
	eatqp2_sh	1	0.96-1.03	7.76E-01	ns	0.84	0.69-1.02	7.34E-02	1.37	1.12-1.69	2.62E-03	0	-0.05	0.86	<2e-16

© 2022 Lee et al. *JAMA Psychiatry*.

eTable 15. Univariate logistic regression analysis results of 30 predictors on SA outcome. Before conducting multivariate analyses, we assessed association of individual predictors with SA using univariate logistic regression. Notations: sa_prs (quantitative, suicide attempt PRS), mdd_prs (quantitative, major depression PRS), adhd_prs (quantitative, ADHD PRS), eatqp2.fr (quantitative, frustration) eatqp2.ag (quantitative, aggression), cbcl_scr_syn_rulebreak_t (quantitative, rule-breaking behavior), cbcl_scr_syn_ aggressive _t (quantitative, aggressive behaviors), cbcl_scr_syn_thought_t (quantitative, thought problems), cbcl_scr_syn_social_t (quantitative, social problems), cbcl_scr_syn_attention_t (quantitative, attention problems), eatqp2.fe (quantitative, fear), cbcl_scr_syn_somatic_t (quantitative, somatic complaints), eatqp2.dp (quantitative, child temperament - depressive mood), cbcl_scr_syn_anxdep_t (quantitative, anxious depresion), cbcl_scr_syn_withdep_t (quantitative, withdrawal depression), eatqp2.ac (quantitative, child temperament – activation control), eatqp2.hi (quantitative, child temperament – high-intensity pleasure/surgency), eatqp2.af (quantitative, child temperament - affiliation), eatqp2.sh (quantitative, child temperament - shy), eatqp2.at (quantitative, child temperament - attention), eatqp2.ic (quantitative, child temperament – inhibitory control), parental.education.college (binary, whether parents engaged/completed college education), household.income (ordinal, 4 categories bases on incomes), parental_scd_p_any (binary, whether any parent has a history of suicide), parental_ma_p_any (binary, whether any parent has a history of mental/emotional problems), and parental_dprs_p_any (binary, whether any parent has a history of depression).

predictor	correlation	cor.p	bonf.p	OR	L95	H95	P	bonf.P	Nagelkerke. R2 (%)
adhd_prs	0.04	2.37E-02	7.10E-01	1.32	1.04	1.69	2.46E-02	2.46E-02	0.88
mdd_prs	0.06	9.78E-04	**2.93E-02**	1.52	1.18	1.95	9.92E-04	9.92E-04	1.91
single.parents	0.09	2.56E-07	**7.68E-06**	3.48	2.1	5.76	1.22E-06	1.22E-06	3.59
parental.no.education.college	0.09	3.88E-07	**1.16E-05**	0.3	0.18	0.49	1.54E-06	1.54E-06	3.72
poverty	0.05	6.67E-03	2.00E-01	3.12	1.32	7.41	9.83E-03	9.83E-03	0.88
household.income	0.08	2.30E-06	**6.90E-05**	0.6	0.48	0.75	4.84E-06	4.84E-06	3.47
cbcl_scr_syn_aggressive_t	0.18	1.21E-22	**3.64E-21**	1.74	1.52	1.99	2.71E-16	2.71E-16	9
cbcl_scr_syn_anxdep_t	0.19	8.25E-28	**2.48E-26**	1.99	1.72	2.3	3.95E-20	3.95E-20	11.97
cbcl_scr_syn_attention_t	0.14	8.86E-15	**2.66E-13**	1.68	1.45	1.94	2.90E-12	2.90E-12	6.52
cbcl_scr_syn_rulebreak_t	0.17	1.11E-20	**3.33E-19**	1.73	1.51	1.98	1.29E-15	1.29E-15	8.46
cbcl_scr_syn_social_t	0.19	1.98E-26	**5.95E-25**	1.87	1.62	2.15	2.46E-18	2.46E-18	10.74
cbcl_scr_syn_somatic_t	0.1	2.03E-08	**6.08E-07**	1.67	1.38	2.01	1.01E-07	1.01E-07	4.29
cbcl_scr_syn_thought_t	0.17	5.64E-21	**1.69E-19**	2.05	1.73	2.43	8.53E-17	8.53E-17	10.37
cbcl_scr_syn_withdep_t	0.15	9.79E-18	**2.94E-16**	1.77	1.53	2.05	1.56E-14	1.56E-14	7.97
eatqp2.ac	-0.02	1.68E-01	1.00E+00	0.84	0.67	1.07	1.61E-01	1.61E-01	0.33
eatqp2.af	-0.03	6.04E-02	1.00E+00	0.79	0.63	1	4.98E-02	4.98E-02	0.64
eatqp2.ag	0.1	8.76E-08	**2.63E-06**	1.84	1.47	2.31	1.30E-07	1.30E-07	4.67
eatqp2.at	0	8.89E-01	1.00E+00	0.98	0.77	1.25	8.59E-01	8.59E-01	0.01
eatqp2.dp	0.15	4.31E-17	**1.29E-15**	2.56	2.03	3.23	2.84E-15	2.84E-15	10.97
eatqp2.fe	0.1	2.71E-08	**8.13E-07**	1.95	1.54	2.49	5.07E-08	5.07E-08	5.17
eatqp2.fr	0.08	4.62E-06	**1.39E-04**	1.8	1.4	2.31	5.17E-06	5.17E-06	3.7
eatqp2.hi	0.06	1.81E-03	5.43E-02	0.69	0.55	0.87	1.91E-03	1.91E-03	1.62
eatqp2.ic	0.06	1.25E-03	**3.74E-02**	1.49	1.17	1.9	1.25E-03	1.25E-03	1.79
eatqp2.sh	-0.02	2.92E-01	1.00E+00	0.88	0.7	1.11	2.83E-01	2.83E-01	0.19
parental_dprs_p_any	0.08	2.50E-05	**7.51E-04**	3.32	1.84	6	6.76E-05	6.76E-05	2.21
parental_ma_p_any	0.05	3.97E-03	1.19E-01	2.9	1.36	6.18	6.05E-03	6.05E-03	1.02
parental_scd_p_any	0.09	3.15E-07	**9.44E-06**	4.46	2.38	8.36	2.98E-06	2.98E-06	2.84

© 2022 Lee et al. *JAMA Psychiatry.*

eTable 16. Additional analysis of cross-disorder GWAS data. To further test the specificity of SA PRS on children's SA outcome, we examined the latest psychiatric cross-disorder GWAS data from the Psychiatric Genomics Consortium (Cross-Disorder Group of the Psychiatric Genomics Consortium, 2019). Here we conducted two-PRS logistic regression analyses to assess whether the identified association between SA PRSs and children's SA stays significant when PRSs for cross disorder genetic risk shared across 9 psychiatric disorders (PGC-CDG2), were added in the same model. Lifetime suicide attempts (SAs) were used as a binary dependent variable, while SA and PGC-CDG2 PRSs were used as independent variables along with age, sex, and top ten principal components of genetic ancestry as covariates. The analysis results showed that cross-disorder genetic risk does not show independent association with children's SA outcome when examined together with SA PRS in the multivariate logistic regression.

Outcome	Event	Subject No	Case No	PRS1 (SA)				PRS2 (PGC-CDG2)			
				OR	L95	H95	Pvalue	OR	L95	H95	Pvalue
SA	baseline	4344	37	1.24	0.89	1.71	2.00E-01	1.22	0.88	1.68	2.43E-01
SA	year 1	4344	74	1.29	1.02	1.62	3.26E-02	1.20	0.95	1.51	1.25E-01
SA	year 2	4344	102	1.36	1.12	1.66	2.05E-03	1.12	0.92	1.36	2.65E-01

© 2022 Lee et al. *JAMA Psychiatry*.

eTable 17. Logistic regression analysis of non-European sample analysis. Studying diverse populations is imperative for addressing major health disparities in human genomics. Through the first PCA and removing population outliers, we identified 4,344 individuals of European ancestry, 1,364 of African ancestry, 1,682 of Hispanic/South American ancestry, and 81 of East Asian, all un-related. Due to the small number of Asian individuals, we focused on the African and Hispanic American ancestry groups. The second PCA was then performed within each of the ancestry groups to capture genetic substructure within the population. Same as our primary EUR analysis, we tested associations between SA PRSs and SI/SA while adjusting for age, sex, and top ten genetic PCs. This analysis revealed no significant associations of SA PRSs in the two non-European ancestry groups. This null finding is likely to occur because of (1) smaller non-European sample sizes (compared to European descents); and (2) of the fact that we are using European SA GWAS on non-European ABCD samples.

Ancestry	PRS	Outcome	Event	Case No	Odds Ratio	95% Confidence Interval	Pvalue
African American	SA	SA	baseline	28 (2.06%)	1.33	0.92-1.93	1.30E-01
			year1	48 (3.52%)	1.11	0.84-1.48	4.63E-01
			year2	59 (4.33%)	1.13	0.87-1.47	3.43E-01
		SI	baseline	93 (6.83%)	1.03	0.84-1.27	7.72E-01
			year1	158 (11.60%)	1.15	0.97-1.35	1.05E-01
			year2	194 (14.24%)	1.06	0.91-1.24	4.27E-01
Hispanic American	SA	SA	baseline	28 (1.67%)	1.03	0.71-1.49	8.90E-01
			year1	40 (2.38%)	1.01	0.74-1.38	9.43E-01
			year2	54 (3.22%)	1.1	0.84-1.44	4.84E-01
		SI	baseline	89 (5.30%)	1.08	0.87-1.33	4.97E-01
			year1	152 (9.05%)	1.01	0.85-1.19	9.20E-01
			year2	206 (12.27%)	1.01	0.87-1.17	9.02E-01

© 2022 Lee et al. *JAMA Psychiatry*.

eReferencesS

1. Zucker RA, Gonzalez R, Feldstein Ewing SW, et al. Assessment of culture and environment in the Adolescent Brain and Cognitive Development Study: Rationale, description of measures, and early data. *Dev Cogn Neurosci*. Aug 2018;32:107-120. doi:10.1016/j.dcn.2018.03.004
2. Barch DM, Albaugh MD, Avenevoli S, et al. Demographic, physical and mental health assessments in the adolescent brain and cognitive development study: Rationale and description. *Dev Cogn Neurosci*. Aug 2018;32:55-66. doi:10.1016/j.dcn.2017.10.010
3. Auchter A, Hernandez Mejia M, Heyser CJ, Shilling PD, Jernigan TL, Brown SA, Tapert SF, Dowling GJ. A description of the ABCD organizational structure and communication framework. *Dev Cogn Neurosci*. 2018;32:8-15.
4. Kaufman J, Birmaher B, Brent DA, Ryan ND, Rao U. K-SADS-PL. *J Am Acad Child Adolesc Psychiatry*. 2000;39(10)
5. Mullins N, Kang J, Campos AI, et al. Dissecting the Shared Genetic Architecture of Suicide Attempt, Psychiatric Disorders, and Known Risk Factors. *Biol Psychiatry*. Feb 1 2022;91(3):313-327. doi:10.1016/j.biopsych.2021.05.029
6. Garcia C, Salmeron R, Garcia C, Garcia J. Residualization: justification, properties and application. *J App Stat*. 2019;47(11):1990-2010.
7. Howard DM, Adams MJ, Clarke TK, et al. Genome-wide meta-analysis of depression identifies 102 independent variants and highlights the importance of the prefrontal brain regions. *Nat Neurosci*. Mar 2019;22(3):343-352. doi:10.1038/s41593-018-0326-7
8. Demontis D, Walters RK, Martin J, et al. Discovery of the first genome-wide significant risk loci for attention deficit/hyperactivity disorder. *Nat Genet*. Nov 26 2019;51:63-75.
9. Achenbach T. International findings with the Achenbach System of Empirically Based Assessment (ASEBA): applications to clinical services, research, and training. *Child Adolesc Psychiatry Ment Health*. 2019;13(1):1.
10. Ellis L, Rothbart MK. Revision of the early adolescent temperament questionnaire. 2001:
11. Auton A, Abecasis GR, Altshuler DM, et al. A global reference for human genetic variation. *Nature*. 2015/10/01 2015;526(7571):68-74. doi:10.1038/nature15393
12. Das S, Forer L, Schonherr S, et al. Next-generation genotype imputation service and methods. *Nat Genet*. Oct 2016;48(10):1284-1287. doi:10.1038/ng.3656
13. Howie B, Fuchsberger C, Stephens M, Marchini J, Abecasis GR. Fast and accurate genotype imputation in genome-wide association studies through pre-phasing. *Nat Genet*. Jul 22 2012;44(8):955-9. doi:10.1038/ng.2354
14. McCarthy S, Das S, Kretzschmar W, et al. A reference panel of 64,976 haplotypes for genotype imputation. *Nat Genet*. Oct 2016;48(10):1279-83. doi:10.1038/ng.3643
15. Chang CC, Chow CC, Tellier LC, Vattikuti S, Purcell SM, Lee JJ. Second-generation PLINK: rising to the challenge of larger and richer datasets. *GigaScience*. 2015;4(1)doi:10.1186/s13742-015-0047-8
16. Choi SW, O'Reilly PF. PRSice-2: Polygenic Risk Score software for biobank-scale data. *Gigascience*. Jul 1 2019;8(7)doi:10.1093/gigascience/giz082
17. Friedman JH, Hastie T, Tibshirani R. Regularization Paths for Generalized Linear Models via Coordinate Descent. *Journal of Statistical Software*. 02/02 2010;33(1):1 - 22. doi:10.18637/jss.v033.i01
18. *mediation: R Package for Causal Mediation Analysis*. Version 3.0. 2013. http://CRAN.R-project.org/package=mediation.
19. *R: A Language and Environment for Statistical Computing*. R Foundation for Statistical Computing; 2020. https://www.R-project.org/
20. Nagelkerke N. A note on a general definition of the coefficient of determination. *Biometrika*. 1991;78:691-692.

© 2022 Lee et al. *JAMA Psychiatry.*
